# Active compensation for changes in *TDH3* expression mediated by direct regulators of *TDH3* in *Saccharomyces cerevisiae*

**DOI:** 10.1371/journal.pgen.1011078

**Published:** 2023-12-13

**Authors:** Pétra Vande Zande, Mohammad A. Siddiq, Andrea Hodgins-Davis, Lisa Kim, Patricia J. Wittkopp

**Affiliations:** 1 Department of Molecular, Cellular, and Developmental Biology, University of Michigan, Ann Arbor, Michigan, United States of America; 2 Department of Ecology and Evolutionary Biology, University of Michigan, Ann Arbor, Michigan, United States of America; University of Georgia, UNITED STATES

## Abstract

Genetic networks are surprisingly robust to perturbations caused by new mutations. This robustness is conferred in part by compensation for loss of a gene’s activity by genes with overlapping functions, such as paralogs. Compensation occurs passively when the normal activity of one paralog can compensate for the loss of the other, or actively when a change in one paralog’s expression, localization, or activity is required to compensate for loss of the other. The mechanisms of active compensation remain poorly understood in most cases. Here we investigate active compensation for the loss or reduction in expression of the *Saccharomyces cerevisiae* gene *TDH3* by its paralog *TDH2*. *TDH2* is upregulated in a dose-dependent manner in response to reductions in *TDH3* by a mechanism requiring the shared transcriptional regulators Gcr1p and Rap1p. *TDH1*, a second and more distantly related paralog of *TDH3*, has diverged in its regulation and is upregulated by another mechanism. Other glycolytic genes regulated by Rap1p and Gcr1p show changes in expression similar to *TDH2*, suggesting that the active compensation by *TDH3* paralogs is part of a broader homeostatic response mediated by shared transcriptional regulators.

## Author summary

Living things have a remarkable ability to modify their molecular and cellular processes to reduce the negative impacts of genetic and environmental perturbations. Here, we examine how cells of baker’s yeast respond to mutations changing the expression of a key metabolic gene, which affects cell division rates (i.e., fitness). We find that the expression of other metabolic genes, including a gene with ancestrally similar sequence, function, and regulation (a paralog), changes its expression in ways complementary to the mutated gene, reducing the impact of the mutation on fitness. This type of compensatory expression change has also been reported for other genes, but the molecular mechanisms behind such changes are generally unknown. In this case, we find that upregulation of the paralog is mediated by transcription factors that also directly regulate the mutated gene. This observation suggests that ancestrally shared regulation of paralogous genes can play a key role in homeostasis and might help maintain genes in the genome following gene duplication.

## Introduction

Biological systems are often robust to genetic and environmental perturbations [1,2]. This robustness is conferred in part by the presence of multiple genes in the genome with overlapping functions [2–4]. Such genes often arise through duplication events that give rise to two or more paralogous genes [5,6]. As described in Diss et al. [7], paralogous genes can contribute to phenotypic robustness through either passive or active mechanisms. In passive paralogous compensation, the normal activity of one of the paralogs is sufficient to minimize the phenotypic impact of losing the activity of the other paralog. By contrast, active paralogous compensation occurs when the activity of one paralog changes in response to loss of activity of the other paralog, reducing the phenotypic impact of this loss. For example, a gene may respond to loss of a paralogous gene’s function by increasing its expression level, producing more protein capable of performing the function of the mutated gene.

Multiple examples of active compensation by upregulation of a paralog have been identified [8–13], but the molecular mechanisms responsible for such transcriptional compensation remain largely unknown. One notable exception is loss of the *CLV1* receptor kinase in *Arabidopsis thaliana*, which is compensated for by the upregulation of paralogous receptor kinases *BAM1*, *BAM2*, and *BAM3*. Under normal circumstances the *BAM* genes are negatively regulated by *CLV1*, and loss of *CLV1* removes this transcriptional repression, resulting in upregulation of the *BAM* genes that compensates for the loss of *CLV1* [14]. This exact mechanism of active compensation for loss of *CLV1* is not conserved in tomato or maize, but other steps in the *CLV* signaling pathway show evidence of active or passive compensation by paralogs within these species [11]. For example, in tomato, upregulation of *SlCLE9*, the closest paralog to *SlCLV3*, in response to loss of *SlCLV3* reduces the phenotypic impact of the *SlCLV3* mutation, although the mechanism causing this upregulation is unclear [11].

Large-scale synthetic genetic interaction studies in the baker’s yeast *Saccharomyces cerevisiae* have also shown that paralogs with overlapping function are frequently able to compensate for each other [15,16]. Up-regulation of paralogous genes with overlapping functions when one paralog is deleted has been reported in *S*. *cerevisiae*, and paralogs with partially overlapping regulatory motifs are more likely to be dispensable than those without overlap suggesting compensation for their loss [17]. A proposed model posits that paralogous enzymes that catalyze the same metabolic step and are regulated by the same transcription factors may act via active compensation. In this framework, accumulation of a metabolic substrate due to reduction in activity of one paralog would trigger feedback mechanisms that increase the activity of shared transcriptional regulators, which in turn cause upregulation of the other paralog, and thus active compensation [17]. There are many examples of feedback circuits from yeast to mammals with the potential to function this way, making the model potentially of wide relevance to many biological systems [18]. To the best of our knowledge, however, the proposed dependency on a shared regulator for active compensation by upregulation of paralogous genes has yet to be demonstrated empirically.

The *Saccharomyces cerevisiae TDH1*, *TDH2*, and *TDH3* genes are paralogs with overlapping protein function and partially overlapping regulation that might make them likely to show active compensation. All three of these proteins act as glyceraldehyde-3-phosphate dehydrogenases (GAPDHs) [19,20], catalyzing a central step in both glycolysis and gluconeogenesis. The *TDH2* and *TDH3* proteins are most similar to each other, retaining 94% amino acid sequence identity [21,22], whereas the *TDH1* and *TDH3* proteins have 89% amino acid sequence identity [22,23]. *TDH2* and *TDH3* are expressed during exponential growth, with *TDH3* expressed at a much higher level, while *TDH1* is expressed primarily during stationary phase [24,25]. The divergence in expression patterns and levels, as well as differences in the sensitivity of *TDH1* to *trans*-regulatory mutations [26], indicates divergence in underlying regulatory control of the paralogs, particularly for *TDH1*. Deletion of *TDH3* reduces fitness to ~93–98% of wild type [27,28] whereas deletion of *TDH1* or *TDH2* alone has little to no effect [28]. Deletion of *TDH1* and *TDH3* together does not exacerbate the fitness defect of deletion of *TDH3* alone; however, deletion of *TDH2* and *TDH3* together shows a strong negative interaction, with growth at only 20% of wild type levels [28]. This nonadditive impact on fitness suggests that the functional overlap of *TDH2* and *TDH3* allows *TDH2* to help compensate for loss of *TDH3*.

Here, we investigate the molecular mechanisms responsible for this compensation. We find that expression of *TDH2* is upregulated when *TDH3* expression is reduced, and downregulated when *TDH3* expression is increased, suggesting that *TDH2* provides active compensation for changes in *TDH3* expression. We show that this active compensation requires functional transcription factor proteins Rap1p and Gcr1p, which directly regulate *TDH3*, and requires binding sites for Gcr1p. We also observe upregulation of *TDH1* when *TDH3* expression is reduced, but this upregulation seems to be independent of Gcr1p, suggesting that there are differences in the molecular mechanisms causing upregulation of the two paralogs. This involvement of Rap1p and/or Gcr1p in the upregulation of *TDH2* provides empirical support for the model proposed by Kafri et al. [17] in which active compensation by paralogous genes is facilitated by one or more shared regulators and feedback loops. But this upregulation is not limited to paralogous genes; we also see upregulation of other genes regulated by Gcr1p and Rap1p that encode proteins that function in the same metabolic pathway. These results suggest that active compensation for changes in *TDH3* expression via upregulation of paralogs is not a specific regulatory program, but rather part of a general activation of the glycolytic regulon. Consequently, this study shows how shared regulators controlling expression of paralogs with overlapping function can provide mutational robustness through active compensation that contributes to homeostasis.

## Results

### Active compensation for loss of TDH3 by TDH2

**Fig 1:**
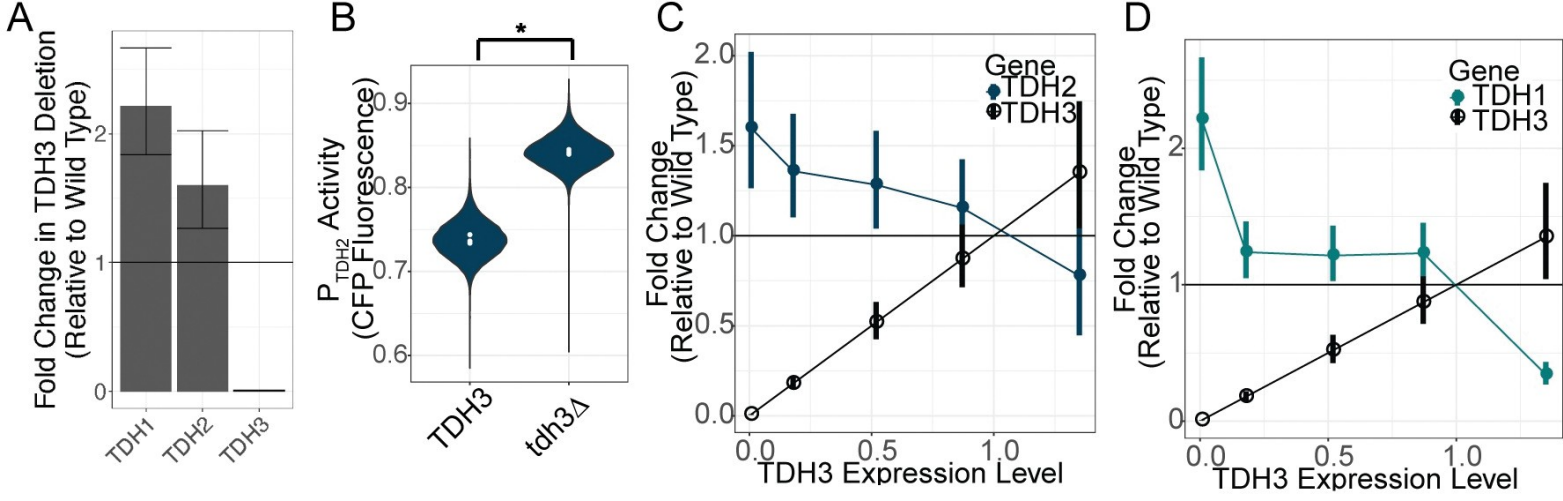
*TDH1* and *TDH2* are upregulated in response to reductions in *TDH3* expression. (A) Changes in expression of *TDH1*, *TDH2*, and *TDH3* in response to the deletion of *TDH3* are shown, measured as fold change in expression relative to a wild type. Error bars represent one standard error of the mean. Statistical significance of expression changes was assessed using Wald tests in DESeq2, with the P-value for *TDH1* = 2x10^-5^, *TDH2* = 0.04, and *TDH3* = 7x10^-107^. (B) Fluorescence normalized to cell size (arbitrary units) is shown for a strain bearing a *P*_*TDH2*_:*CFP-TDH2* fusion protein with *TDH3* intact (TDH3) or deleted (tdh3Δ). Each violin plot represents data from four biological replicates, each containing 15,000 singlet cells measured by flow cytometry. White points indicate medians from each of the four replicates. Expression in the tdh3Δ strain is significantly higher than the TDH3 strain (one sided t-test p-value = 2.23x10^-7^) (C) Changes in expression of *TDH3* and *TDH2* are shown for strains with *cis*-acting mutations causing 0%, 20%, 50%, 85%, and 135% of wild type *TDH3* expression. Error bars show one standard error of the mean. (D) Changes in expression of *TDH3* and *TDH1* are shown for strains with *cis*-acting mutations causing 0%, 20%, 50%, 85%, and 135% of wild type *TDH3* expression. Error bars show one standard error of the mean. RNA-sequencing data in panels A, C, and D from [29]. As described previously, data from each strain is composed of 2 (*TDH3* deletion) or 4 (others) biological replicates.

To determine whether the compensation for loss of *TDH3* activity by *TDH2* might be mediated by changes in their expression, we examined *TDH2* expression in a *TDH3* deletion strain of *S*. *cerevisiae* (*tdh3Δ)* previously analyzed using RNA-seq [29]. We found that *TDH2* showed significantly higher expression in the *tdh3Δ* strain than in the unmutated wild-type strain (Fig 1A, Wald test P-value for *TDH2* = 0.04). To determine whether this upregulation also affects protein abundance, we engineered strains in which a cyan fluorescent protein (CFP) was fused to the native *TDH2* protein and assayed expression level of this fusion protein using flow cytometry. We found that fluorescence increased upon deletion of *TDH3*, indicating an increase in Tdh2p (Fig 1B, one sided t-test p-value = 2.23x10^-7^). To determine whether the degree of upregulation correlates with the extent to which *TDH3* expression is altered, we used additional RNA-seq data from Vande Zande *et al* [29] to examine *TDH2* expression in strains of *S*. *cerevisiae* carrying changes in the *TDH3* promoter that cause more moderate alterations in *TDH3* expression. Three of these strains carry a single point mutation in the *TDH3* promoter that drives either 20%, 50%, or 85% of wild-type *TDH3* expression [29]. A fourth strain carries a duplication of the *TDH3* gene with each copy carrying a single promoter mutation reducing expression levels from each promoter, resulting in a strain expressing *TDH3* at 135% of wild-type levels. We found that *TDH2* expression was negatively correlated with *TDH3* expression among these strains, with *TDH2* showing both increased expression when *TDH3* expression was decreased and decreased expression when *TDH3* expression was increased (Fig 1C). We also examined the expression levels of the *TDH3* paralog *TDH1* and found that *TDH1* is also upregulated in the *tdh3Δ* deletion mutant (Fig 1A, Wald test P-value for *TDH1* = 2x10^-5^). Unlike *TDH2*, however, *TDH1* showed more of a threshold-like relationship with *TDH3* expression: *TDH1* expression was strongly increased in the *TDH3* null strain, but only mildly (and similarly) increased in the mutant strains expressing *TDH3* at 20%, 50%, and 85% of wild-type levels (Fig 1D). Like *TDH2*, *TDH1* expression decreased in the strain overexpressing *TDH3* (Fig 1D). Differences in the expression changes observed for *TDH1* and *TDH2* in these *TDH3* mutants are consistent with divergence in the regulation of *TDH1* and *TDH2* (S1 Fig). Taken together, these data provide evidence of active compensation when *TDH3* expression is altered, with expression of its paralog *TDH2* changing in ways expected to minimize the impacts of these *TDH3* mutations on fitness.

### Active compensation might be caused by direct regulators of TDH3

**Fig 2:**
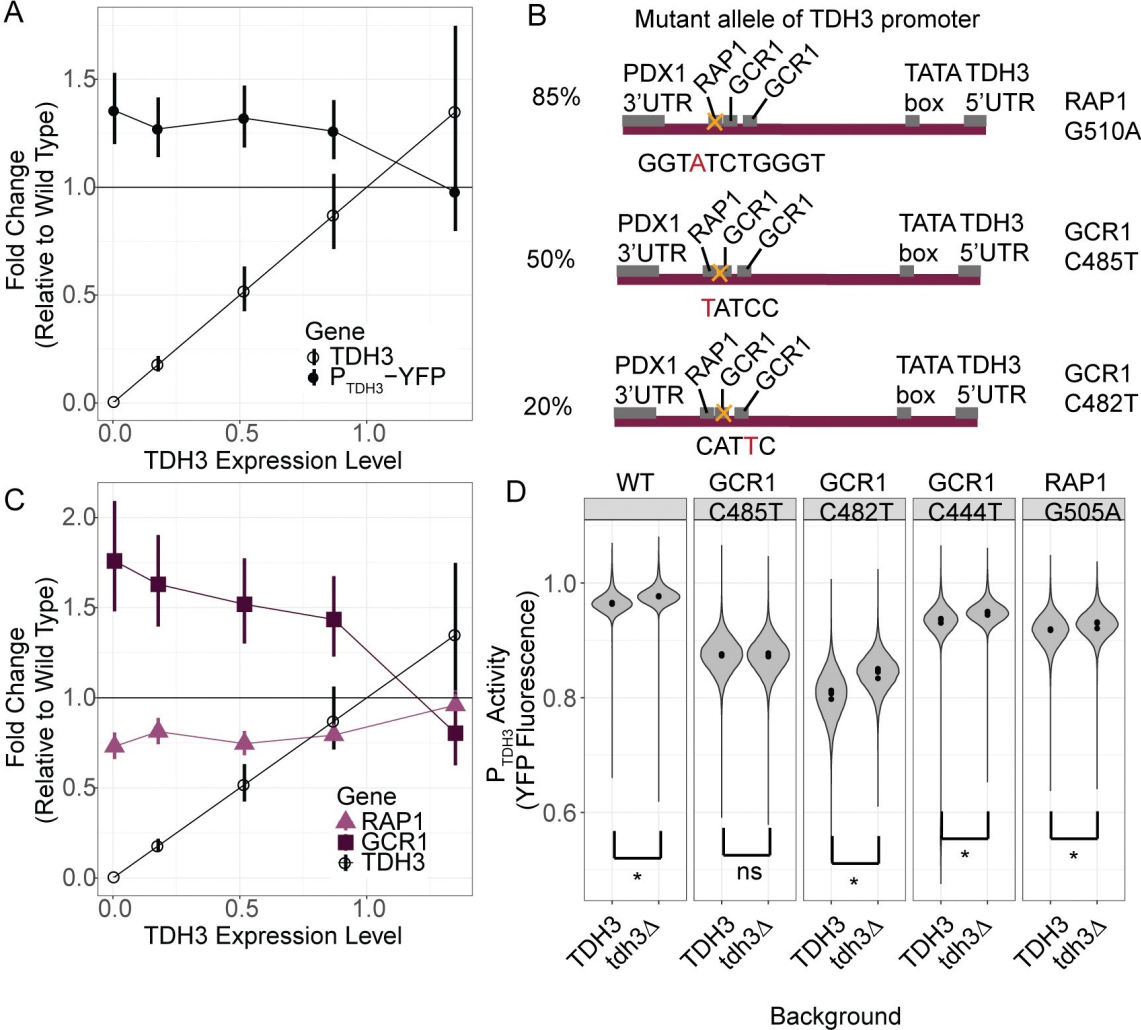
Feedback regulating TDH3 expression is mediated by Gcr1p TFBSs. (A) Changes in expression of *TDH3* and a reporter gene with a wild type *TDH3* promoter driving expression of YFP (*P*_*TDH3*_*-YFP*) are shown for strains with *cis*-acting mutations causing 0%, 20%, 50%, 85%, and 135% of wild type *TDH3* expression. Error bars show one standard error of the mean. (B) Schematics and sequences of the *TDH3* promoter in mutant strains bearing mutations in binding sites for Rap1p and Gcr1p at a distance of 510, 485, and 482 nucleotides upstream of the *TDH3* ATG that result in *TDH3* expression levels of 20%, 50%, and 85% relative to wild type are shown. No schematic is shown for the mutant strain expressing *TDH3* expression at 135% of wild type levels; it contains two copies of the *TDH3* gene separated by a copy of the *URA3* gene, with both copies of *TDH3* containing a mutation in the binding site for Rap1p at a distance of 505 nucleotides upstream of the ATG (GGTGTCTGaGT). (C) Changes in expression of *RAP1*, *GCR1*, and *TDH3* are shown for strains with *cis*-acting mutations in the *TDH3* promoter causing 0%, 20%, 50%, 85%, and 135% of wild type *TDH3* expression, measured as fold change in expression relative to a wild type. Error bars represent one standard error of the mean. RNA-sequencing data in panels A and C are from [29]. (D) Fluorescence normalized to cell size (arbitrary units) is shown for a strains bearing a *P*_*TDH3*_:*YFP* construct with point mutations in transcription factor binding sites of either Gcr1p or Rap1p, each with *TDH3* intact (TDH3) or deleted (tdh3Δ). These include two of the three mutant promoter alleles shown in (B), one mutant allele at the 505 position in the Rap1p TFBS resulting in ~70% expression, plus a mutant allele containing a mutation 444 nucleotides upstream of the TDH3 start codon, which affects the GCR1 binding site closer to the TATA box shown in (B). Each violin plot consists of four biological replicates each containing 15,000 singlet cells measured by flow cytometry. Points indicate medians of each of the four replicates. Asterisks indicate significantly higher expression in the tdh3dΔ strain as compared to the matched TDH3 strain (one-sided t-test for median fluorescence p-values from left to right are: 8.7x10^-6^, 0.78, 1.3x10^-4^, 6.7x10^-4^, 0.014).

For historical reasons [27], the control strain and *TDH3* mutant strains profiled for expression using RNA-seq in Vande Zande et al [29] all carried a reporter gene composed of the wild-type *TDH3* promoter allele driving expression of a yellow fluorescent protein (*P*_*TDH3*_*-YFP*). Surprisingly, we found that expression of this reporter gene was increased when native *TDH3* expression was decreased by mutations in its promoter and not by the duplication of *TDH3* with promoter mutations causing over-expression of *TDH3* (Fig 2A). This negative correlation between expression of the native *TDH3* gene harboring *cis*-acting mutations and expression driven by a wild-type allele of the *TDH3* promoter suggests that factors regulating expression of *TDH3* itself might be involved in the mechanism of active compensation.

The transcription factors Rap1p and Gcr1p regulate expression of *TDH3* [30,31] as well as expression of other glycolytic genes, including *TDH1* and *TDH2* [32–35]. In fact, the mutations altering expression of *TDH3* in the mutant strains expressing *TDH3* at 20%, 50%, and 85% of wild-type expression levels all altered either Rap1p or Gcr1p binding sites in the *TDH3* promoter (Fig 2B, [27,29]). We thus wondered whether transcription of *RAP1* and/or *GCR1* was changed in the strains with *TDH3* promoter mutations. Using the same RNA-seq dataset described above, we found that *GCR1* was upregulated linearly in response to reductions in *TDH3* expression caused by mutations in the *TDH3* promoter whereas expression of *RAP1* was not (Fig 2C). If anything, expression of *RAP1* was slightly and similarly reduced in all mutants with reduced *TDH3* expression (Fig 2C).

We next tested whether the Rap1p and Gcr1p transcription factor binding sites (TFBS) were necessary for the upregulation of the reporter driven by the *TDH3* promoter upon reduction in *TDH3* expression. We engineered strains with mutations in either Rap1p or Gcr1p TFBSs in the *TDH3* promoter driving YFP expression (S1 Table, S1 File). We then deleted the native *TDH3* locus in these backgrounds and found that fluorescence did not significantly increase upon reduction in *TDH3* in one of the Gcr1p TFBS mutants (Fig 2D, one-sided t-test for median fluorescence p-values from left to right are: 8.7x10^-6^, 0.78, 1.3x10^-4^, 6.7x10^-4^, 0.014), showing that the specific nucleotides in this Gcr1p binding site are necessary for compensatory upregulation via the *TDH3* promoter.

### Mutations in Rap1p and Gcr1p disrupt compensatory expression changes of TDH2

**Fig 3:**
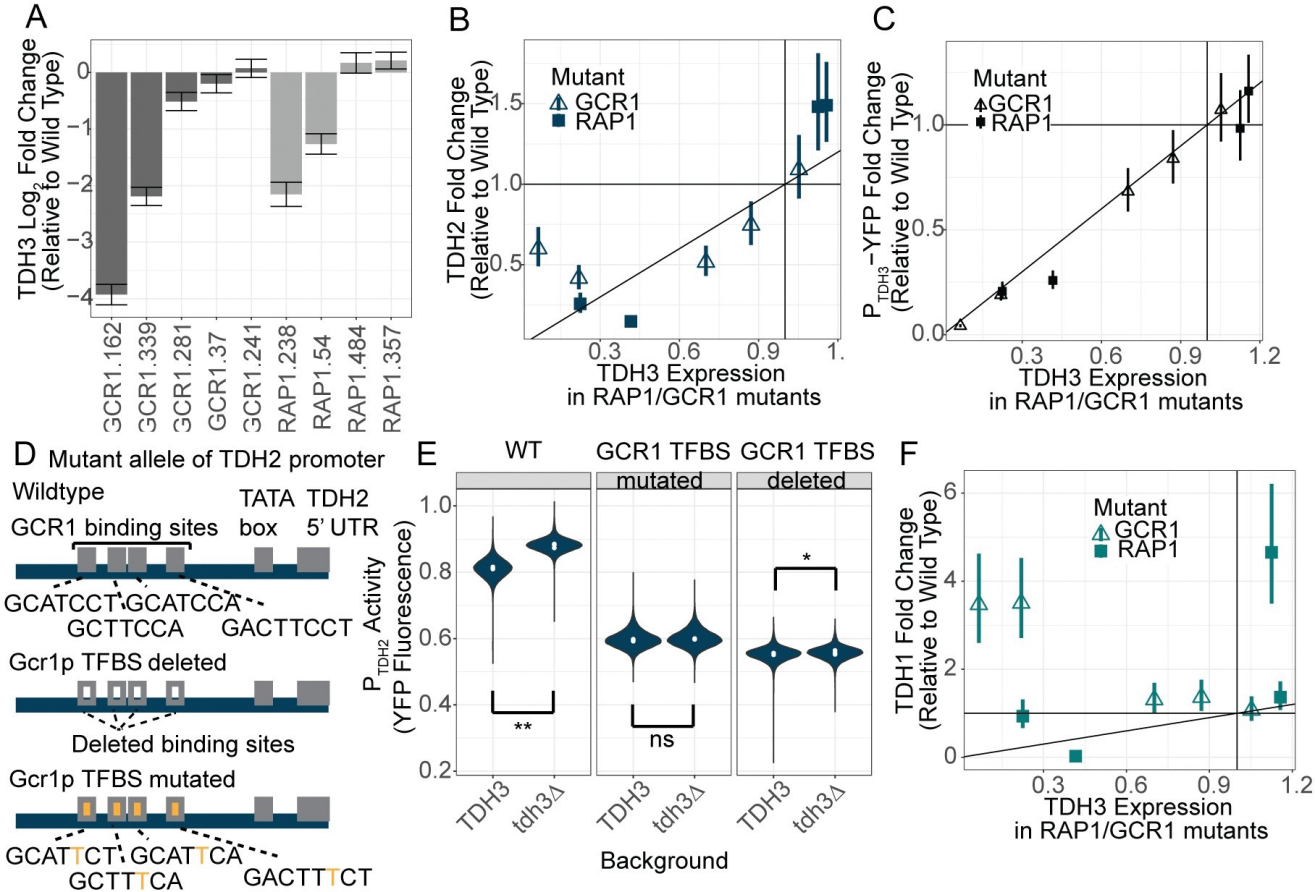
Active compensation by TDH2 is mediated by RAP1 and/or GCR1. (A) Changes in expression of *TDH3* in response to various mutations in either *GCR1* (dark grey) or *RAP1* (light grey), measured as log_2_ fold change in expression relative to a wild type. Specific mutation identities in each strain are described in S1 Table. Error bars represent one standard error of the mean. (B, C) Fold changes in expression of *TDH3* and *TDH2* (B) or a reporter gene with a wild type *TDH3* promoter driving expression of YFP (*P*_*TDH3*_*-YFP*) (C), are shown for strains with mutations in either the *RAP1* (squares) or *GCR1* (empty triangles) coding sequences. Error bars show one standard error of the mean. RNA-sequencing data are from [29]. As described previously, RNA-sequencing data for each strain consists of 4 biological replicates. (D) Schematics of the *TDH2* promoter driving YFP, indicating Gcr1p TFBSs that are either deleted or mutated. (E) Fluorescence normalized to cell size (arbitrary units) is shown for strains bearing a *P*_*TDH2*_*-YFP* construct that is wild type, with Gcr1p TFBSs mutated, or with Gcr1p TFBSs deleted (as shown in panel D), each with *TDH3* intact (*TDH3*) or deleted (*tdh3Δ*). Each violin plot consists of four biological replicates each containing 15,000 singlet cells measured by flow cytometry. Points indicate medians of each of the four replicates. (One sided t-test for median fluorescence p-values from left to right are: 5.17x10^-6^, 0.18, 0.027) (F) As in panels B and C, fold changes in expression of *TDH1* is shown for strains with mutations in either *RAP1* or *GCR1* coding sequences.

If Rap1p and/or Gcr1p are involved in the upregulation of *TDH2* upon reduction of *TDH3* expression, we expect that strains with mutations in Rap1p or Gcr1p causing a reduction in *TDH3* expression would not show the same compensatory upregulation of *TDH2* seen in strains with wildtype *Rap1* and *Gcr1* proteins. That is, if the upregulation of *TDH3* paralogs requires Rap1p or Gcr1p, then mutations in these proteins that disrupt their ability to drive *TDH3* expression at wild-type levels should also impair their ability to upregulate expression of other genes in response to reduced *TDH3*. To test this hypothesis, we examined RNA-seq data from 9 mutant strains of *S*. *cerevisiae* each carrying 1–6 mutations in the *RAP1* (4 mutants) or *GCR1* (5 mutants) gene previously shown to affect *TDH3* expression [36]. These data were collected in parallel with the expression data for the *TDH3* mutants [29]. One *GCR1* mutant strain (GCR1.162) carried a single nucleotide deletion resulting in an early stop codon, suggesting it was likely to be a null mutation. This mutant expressed *TDH3* at only 7% of wild-type expression levels (Fig 3A). The other *GCR1* mutant alleles were more likely to be hypo- (GCR1.339, GCR1.281, GCR1.37) or hypermorphs (GCR1.241), causing *TDH3* expression to range from ~22% to ~105% of wild type levels (Fig 3A). *RAP1* null mutants are lethal [37], suggesting that all of the *RAP1* mutants examined were either hypo- or hypermorphs. These *RAP1* mutants showed *TDH3* expression ranging from ~20% to ~115% of wild-type levels (Fig 3A).

Consistent with Rap1p and Gcr1p mediating compensatory changes in paralog gene expression, we found that the *TDH2* gene was not upregulated in either the Rap1p or Gcr1p mutants that decreased *TDH3* expression (Fig 3B). *TDH2* expression was also not reduced in mutants causing overexpression of *TDH3* (Fig 3B). These observations suggest that both Rap1p and Gcr1p are required for the compensatory changes in *TDH2* expression seen in strains carrying mutations in the *TDH3* promoter. Changes in expression of the *P*_*TDH3*_*-YFP* reporter gene seen in the *TDH3* mutants (Fig 2A) were also absent in the *RAP1* and *GCR1* mutants altering *TDH3* expression (Fig 3C), again implying that Gcr1p and Rap1p were required for these changes.

We next tested whether regulation by Gcr1p was required for the compensatory upregulation of *TDH2* by engineering strains with variations of the *TDH2* promoter driving YFP expression, including an unmutated *TDH2* promoter, a *TDH2* promoter in which each putative Gcr1p TFBS was completely deleted, and a *TDH2* promoter bearing single point mutations in each Gcr1p TFBS expected to disrupt Gcr1p binding (Fig 3D). The wild type *TDH2* promoter drove higher expression upon deletion of the native *TDH3* locus, as expected from our RNA-seq data (Fig 3E, one sided t-test for median fluorescence p-value = 5.17x10^-6^). Abolishing all Gcr1p TFBS greatly reduced pTDH2 activity, while point mutations in all Gcr1p TFBS reduced activity to a lesser extent (Fig 3E). In both cases, deletion of *TDH3* failed to induce comparable increases in expression of the *TDH2* promoter (Fig 3E, one sided t-test for median fluorescence p-values = 0.18, 0.027). Therefore, we conclude that the compensatory increase in *TDH2* promoter activity upon deletion of *TDH3* is dependent upon intact Gcr1p TFBS.

Expression of *TDH1*, on the other hand, showed increases in expression in *GCR1* mutants with lowered *TDH3* expression (Fig 2F), suggesting that Gcr1p is not required for the upregulation of *TDH1* in response to reduced expression of *TDH3*. Rap1p might be required for this upregulation, however, because neither of the *RAP1* mutants decreasing *TDH3* expression showed an upregulation of *TDH1* (Fig 3F). These data support a model in which Gcr1p is involved in the active compensation for changes in *TDH3* expression via *TDH2*, but not *TDH1*, with Rap1p involved in the changes in expression of both genes.

### Expression changes are also seen for other, non-paralogous, metabolic genes

**Fig 4:**
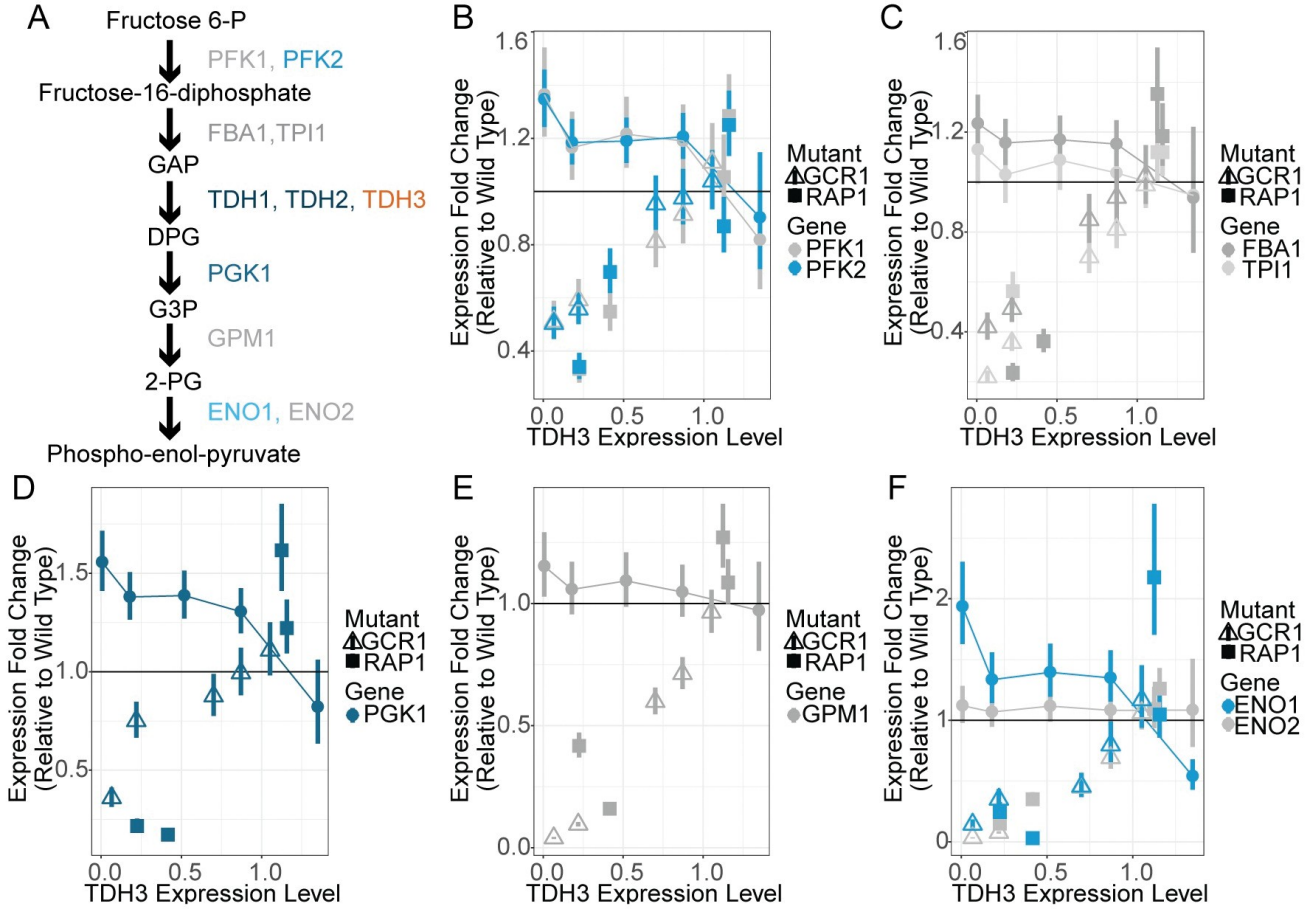
Multiple enzymes in the glycolysis pathway are upregulated upon reduction in TDH3 expression in a RAP1/GCR1 dependent manner. (A) A simple schematic of the glycolytic pathway surrounding the metabolic step catalyzed by TDH1, TDH2, and TDH3, showing other enzymes catalyzing adjacent reactions. Enzymes that are significantly upregulated upon reduction in TDH3 are in blue. Enzymes in this pathway that were not statistically significantly upregulated are shown in grey. Differences in the variance among replicates for PFK1 and PFK2 resulted in PFK2 but not PFK1 being statistically significantly upregulated even though the two genes showed similar magnitudes of upregulation. (B-F) Expression fold changes relative to wild type of the genes PFK1 and PFK2 (B), FBA1 and TPI1 (C), PGK1 (D), GPM1 (E), and ENO1 and ENO2 (F) in yeast strains with varying levels of TDH3 expression due to mutations in the native TDH3 promoter (circles connected by lines), and in the 9 yeast strains with varying levels of TDH3 expression due to mutations in the genes encoding RAP1 (solid boxes) or GCR1 (empty triangles) as estimated by RNA-sequencing data from Vande Zande et al [29]. Error bars are one standard error of the mean.

Rap1p and Gcr1p are transcription factors that regulate expression of many metabolic genes [38,39], thus active compensation for altered *TDH3* expression mediated by Rap1p and Gcr1p might affect more than just genes paralogous to *TDH3*. Indeed, the eight genes encoding enzymes that function in the glycolytic pathway at steps immediately surrounding the step controlled by the TDH proteins have all been annotated as targets of Gcr1p and Rap1p based on either gene expression and/or chromatin immunoprecipitation experiments [33–35]. We therefore examined the expression of these genes (Fig 4A) in the RNA-seq data from *TDH3*, *RAP1*, and *GCR1* mutants described above. We found that each of these genes was upregulated in the *tdh3Δ* null mutant and their expression levels showed an inverse relationship with *TDH3* expression in the other *TDH3* promoter mutants (Fig 4B–4F, circles), although the magnitude of increased expression was variable between genes. The detected upregulation of other glycolytic genes was likely mediated by Rap1p and Gcr1p because none of the aforementioned genes were upregulated in strains bearing mutations in *RAP1* or *GCR1* in response to reduced levels of *TDH3* (Fig 4B–4F, squares and triangles). The similarity of expression patterns between other glycolytic enzymes and *TDH2* suggests that the compensatory upregulation of *TDH2* is part of the larger homeostatic network regulating expression of genes in the glycolytic pathway and is not specific to compensation by paralogs. Activation of the entire pathway is consistent with active compensation for changes in *TDH3* expression being mediated through homeostatic feedback mechanisms involving Gcr1p and Rap1p in place for the regulation of glycolysis.

## Discussion

**Fig 5:**
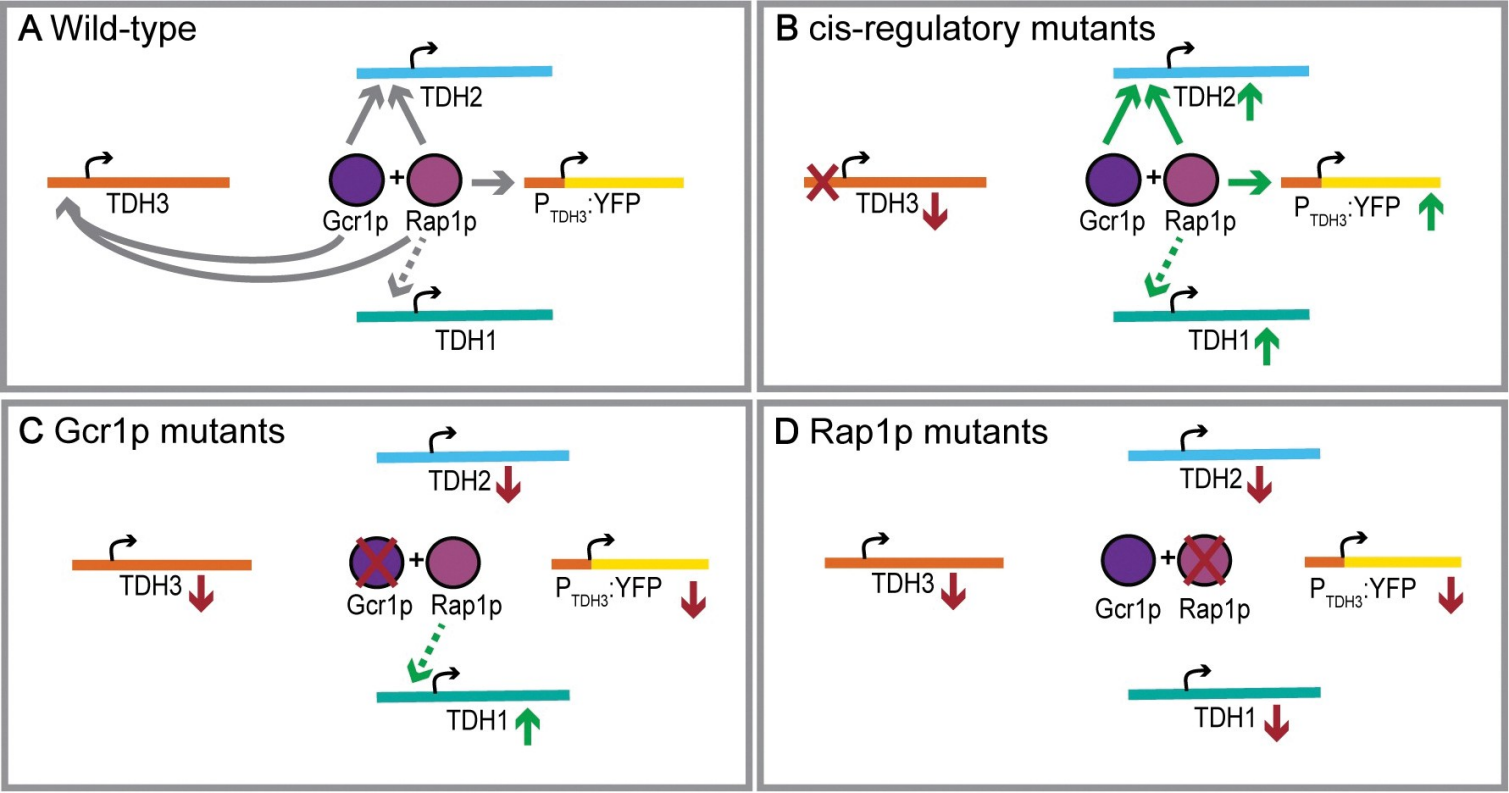
Model for active compensation by feedback and shared regulation. (A) In a wild-type cell, the Gcr1p and Rap1p complex regulate expression levels of *TDH2* and *TDH3*, and Rap1p likely regulates expression of *TDH1*. (B) When the native promoter of *TDH3* is mutated, *TDH3* levels decrease, leading to an upregulation of *TDH2* and a *P*_*TDH3*_*-YFP* reporter gene via Gcr1p and Rap1p, and *TDH1* via Rap1p. (C) When Gcr1p is mutated, levels of all its direct targets are reduced. Lower levels of *TDH3* lead to an upregulation of *TDH1*, possibly via Rap1p. (D) When Rap1p is mutated, levels of all its direct targets are reduced. Despite lower levels of *TDH3* expression, the paralogs are not upregulated due to lack of functional Rap1p.

Many genes with overlapping functions can compensate for each other’s loss, contributing to the genetic robustness of biological systems, but the mechanisms by which this compensation arises, operates, and is maintained over evolutionary time continues to be unclear [40–42]. In this study, we show that changes in *TDH3* expression trigger feedback mechanisms that depend on the activity of transcription factors Rap1p and Gcr1p to offset the effects of these changes. Strains bearing *cis*-regulatory mutations in the *TDH3* promoter that decrease its expression presumably fail to upregulate *TDH3* because the transcription factor binding sites for Rap1p or Gcr1p are disrupted in these alleles (or because the locus is absent in the null mutant), yet expression of other genes regulated by Gcr1p and Rap1p is increased, including the *TDH3* paralogs *TDH2* and *TDH1* and even a reporter gene driven by a wild-type *TDH3* promoter. In other words, reduction in *TDH3* expression results in active compensation by upregulation of its paralog *TDH2* (Fig 5).

The upregulation of *TDH2* by Gcr1p/Rap1p might be achieved by increased expression of the *GCR1* gene in response to reduced *TDH3* expression. Transcriptional upregulation is not the only mechanism of activation of transcription factors [43], but *GCR1* has been shown to be both transcriptionally and post-transcriptionally regulated by glucose availability [44] and we observed increased *GCR1* expression in mutants with decreased *TDH3* expression, demonstrating that this transcription factor is transcriptionally regulated under some circumstances. *RAP1*, on the other hand, performs roles in telomere maintenance and activation of ribosomal protein genes in addition to the activation of glycolytic genes [45,46], and is not known to be transcriptionally responsive to metabolic changes. Because Rap1p and Gcr1p act in a complex to activate target gene expression, with Gcr1p being the major activator of the complex [38], we propose that upregulation of *GCR1* transcription upon reduction in *TDH3* expression is primarily responsible for the upregulation of the Rap1p/Gcr1p complex’s target genes, while still being dependent on functional Rap1p for upregulation of its target genes.

Upregulation of *TDH1* appears to occur via a different mechanism, as indicated by its more threshold-like response to reduction in *TDH3* expression and its upregulation in strains bearing mutations in *GCR1*. These differences in how *TDH1* and *TDH2* respond to reduction in *TDH3* expression may not be surprising given that the expression pattern of *TDH1* has diverged from that of the other two paralogs (S1 Fig, [19]). *TDH1* has been shown to be upregulated under various stress conditions causing slow growth [20], and might therefore be upregulated by a mechanism related to the slower growth of mutants with reduced *TDH3* expression level rather than feedback specifically involving Gcr1p, although it does appear to be at least somewhat dependent on Rap1p function (Fig 3F).

The fact that the upregulation of *TDH2* does not completely eliminate the fitness effect of deleting *TDH3* suggests that either the upregulation of *TDH2* does not produce as much GAPDH activity as the normal expression of the *TDH3* gene (which is consistent with the data shown in Figs 1C and S1) or that there are pleiotropic effects of the compensation mechanism itself, such as the upregulation of other glycolytic enzymes, that causes a fitness cost [47]. Alternatively, the upregulation of other enzymes in the pathway could be beneficial, and any remaining fitness costs due to some divergence in the functions of the *TDH2* and *TDH3* proteins such that they cannot completely compensate for each other. Although *TDH3* is best known for its roles in glycolysis and gluconeogenesis, it has also been implicated in transcriptional silencing [48], RNA-binding [49] and antimicrobial defense [50], functions which may not be able to be compensated for by *TDH2* despite their high levels of protein conservation. More work assessing these ‘non-canonical’ or ‘moonlighting’ [51–53] functions of the GAPDHs in *S*. *cerevisiae* is needed to better understand their relative roles in the cell.

The redundancy of paralogous genes both imparts robustness to biological systems and simultaneously makes them evolutionarily unstable given that mutations in one gene are masked by the presence of the other gene. Yet, paralogous genes with overlapping functions are sometimes maintained over long evolutionary timescales [4,15,16,18,54–58]. Divergence in gene regulation and/or protein function might contribute to the maintenance of all three TDH paralogs over evolutionary time; however, in general, it remains to be seen how often the ability of paralogs to actively compensate for each other and contribute to genetic robustness is actively selected for or simply a side effect of their ancestrally shared regulators with sensitivity to feedback mechanisms. Decoding the molecular mechanisms responsible for active compensation among paralogous genes in other systems will help address this issue, revealing how living systems can thrive despite the inevitable changes in the environment and their genotype.

## Materials and methods

### Strains used in this study

The *S*. *cerevisiae* strains used in this study are haploid strains derived from S288C and include the 5 *cis-*regulatory mutants affecting expression of *TDH3* containing changes in the *S*. *cerevisiae TDH3* promoter and the 9 *trans*-regulatory mutants affecting expression of *TDH3* that each carry 1–6 mutations in either the *RAP1* or *GCR1* gene described in Vande Zande et al. [29]. Construction of the *cis-*regulatory mutant strains, including the *tdh3Δ* strain, is described inx2 [27], and construction of the strains bearing mutations in the *RAP1* or *GCR1* genes is described in [36].

Reporter strains were constructed by fusing either the *TDH1*, *TDH2*, or *TDH3* promoters to VenusYFP and engineering this construct into the *HO* locus of a haploid S288C strain, as described in [59]. Variants of the *TDH3* and *TDH2* promoters were constructed similarly: promoter alleles with desired mutations in the Gcr1p or Rap1p binding sites were cloned upstream of the YFP coding sequence in a plasmid with homology arms to the *HO* locus using Gibson assembly. The constructs were subsequently amplified using PCR and engineered into the *HO* locus using CRISPR-Cas9, following methods described in Laughery et al. [60]. Proper integration into the HO locus was verified using Sanger Sequencing. The *CFP-TDH2* fusion strain was constructed using PCR SOE (Splicing by Overlap Extension) to generate a DNA fragment with CFP fused to the 5’ end of *TDH2*. CRISPR-Cas9 was used to introduce a double-stranded break at the 5’ end of the native *TDH2* locus [60]. Insertion of the *CFP-TDH2* DNA fragment containing homology arms to the *TDH2* promoter and gene interrupted the gRNA PAM recognition site. Then, correct insertion was confirmed by Sanger sequencing of two amplicons spanning the edited locus. Sequences for each sgRNA and inserted construct are available in S1 File.

The collection numbers and specific mutations in each strain, as well as their impacts on *TDH3* expression, are detailed in S1 Table.

### Gene expression data

RNA-sequencing data presented in this paper is a subset of the data described Vande Zande et al. [29] and are available at GEO accession GSE175398. That dataset consists of RNA-sequencing data for *cis-*regulatory mutants and a larger set of *trans-*regulatory mutants affecting *TDH3* expression. Details of data collection and processing are available in [29] and are summarized here. Briefly, yeast cells were grown to mid log phase in glucose media, pelleted, and frozen at -80C. polyA RNA was extracted from frozen cell pellets using oligodT magnetic beads. RNA libraries were prepared for sequencing using a ⅓ volume TruSeq RNA Sample Preparation v2 kit (Illumina) and sequenced on a HiSeq 4000 by the University of Michigan Sequencing Core. Each genotype (all mutants and non-mutated reference strains) was assayed in quadruplicate with each replicate consisting of a unique random array of genotypes and controls in a 96 well plate.

### Measures of fluorescence changes over during population growth

Strains with different YFP reporter genes (driven by either *P*_*TDH1*_, *P*_*TDH2*_, or *P*_*TDH3*_) were patched from glycerol stocks onto YPG agar media (10g/L yeast extract, 20g/L peptone, 20g/L agar, 20% glycerol) and grown at 30C for 2–3 days. They were subsequently grown in YPD (10g/L yeast extract, 20g/L peptone, 20% dextrose) liquid culture for 48–72 hours, until all strains had reached stationary phase. The saturated strains were then diluted into 96 well plates, with 5ul of saturated culture added to 195ul of YPD. The plates were then incubated at 30C with shaking in a Synergy H1 plate reader. Culture growth and reporter gene expression were characterized by recording OD660 and YFP fluorescence readings, respectively, at 20-minute intervals for 48 hours. Three replicates were measured for each genotype using this method.

### Flow cytometry

All flow cytometry data is deposited at flowrepository.org and publicly accessible at http://flowrepository.org/id/FR-FCM-Z72G. Strains bearing fluorescent proteins were patched from glycerol stocks onto 5-FOA agar media (0.67% YNB, 0.2% SC-uracil dropout mix, 2% glucose, 50ug/mL uracil, 0.1% 5-FOA) and grown at 30C for 3 days then stored briefly at 4C. Strains were revived by inoculation in liquid YPD (10g/L yeast extract, 20g/L peptone, 20% dextrose) and grown to saturation: 1mL YPD cultures inoculated in 14mL culture tubes were incubated at 30C with 200 rpm shaking for 48h in 4 replicates. Saturated cultures were then back diluted to inoculate cultures for scoring by adding 50uL of saturated culture to 1mL fresh YPD in culture tubes and incubating at 30C with 200 rpm shaking. After 24h growth, strains were diluted for scoring by transferring 20uL saturated culture into 0.5mL phosphate-buffered saline (Thermo Scientific blood bank saline, pH 7.0–7.2 cat#8504). Fluorescence was quantified on a Cytek Aurora analyzer (U1359SP) located at the University of Michigan BRCF Flow Cytometry Core. Cultures were diluted and scored one replicate at a time using autosampling from a 40-tube rack (flow rate: medium, 6 sec data recording delay, 2s agitation every 2 wells). FCS files were exported and analyzed in R (version 4.1.3) using the *flowCore* [61] and *flowClust* [62] packages. Cells were filtered for size using hard gates set on FSC.A and SSC.A values and a clustering-based gate on FSC.A, and then a singlets gate applied. Raw fluorescence units were normalized to cell size by dividing log_10_ of fluorescence in the bandpass filter appropriate for each fluorochrome (CFP: V5 detector 405 excitation, 508/20 emission; YFP: B2 detector: 488 excitation, 525/17 emission) by the log_10_ FSC.A value. All samples were down-sampled to 15,000 singlet cells per sample to equalize the number of cells analyzed for each strain. Each violin plot is composed of all four biological replicates, each consisting of 15,000 single cell events for a total of 60,000 cells. A point at the median of each replicate (4 points total in each violin plot) is superimposed over the violin plots. Medians of each replicate were used for statistical tests for difference of means (Student’s t-test).

### Statistical analysis

All statistical analysis was performed in R, (version 3.5.2). As described in Vande Zande et al. [29], RNA-seq reads were pseudomapped to the *S*.*cerevisiae* transcriptome (Ensemble, release 38, retrieved from http://ftp://ftp.ensemblgenomes.org/pub/release-38/fungi/fasta/saccharomyces_cerevisiae/cdna/), and DeSeq2 [63] was used to estimate log_2_ fold changes and significance values reported in the text. One-sided t-tests for flow cytometry data were performed in R using the base ‘t.test’ function (alternative = “greater”) to compare median fluorescence intensity between reporter strains with intact *TDH3* and *tdh3Δ*. Each comparison consisted of four biological replicates. Code used in the analysis and to generate figures in this manuscript is available at Github and in a permanent zenodo release (URL: https://github.com/pvz22/Compensation_TDH3, Zenodo DOI: 10.5281/zenodo.10223579).

## Supporting information

S1 Table Strains used in this study. Table including all mutant strains of *S*. *cerevisiae* used in this study, including those for which RNA-sequencing data was collected and strains bearing fluorescent protein reporters.
(XLSX)

S1 Fig *TDH1*, *TDH2*, and *TDH3* are expressed at different levels and under different growth conditions. (A) Population growth curves (measured using the optical density (OD) at 660 nm) are shown for strains expressing a yellow fluorescent protein (YFP) driven by *P*_*TDH1*_ (light blue), *P*_*TDH2*_ (dark blue), or *P*_*TDH3*_ (black). All three strains showed similar growth dynamics. Data in (A) was used to demarcate lag phase (red), exponential growth (green), diauxic shift (blue), and respiratory growth (purple) phases for all panels. Three replicates of each strain are shown. (B-D) Fluorescence values normalized to OD660 to account for changes in cell density are plotted across the growth curve for strains expressing YFP driven by *P*_*TDH3*_ (B), *P*_*TDH2*_ (C), and *P*_*TDH1*_ (D). *P*_*TDH3*_ and *P*_*TDH2*_ dynamics are similar across the stages of the growth curve (lag phase in red, exponential growth in green, diauxic shift in blue, and respiratory growth in purple, see panel A), with expression increasing during early exponential growth and then declining, halting during the diauxic shift, and increasing at a slower rate throughout respiratory growth. *P*_*TDH1*_ shows different dynamics; as *TDH2* and *TDH3* begin to decline, *TDH1* begins to increase and does so steadily throughout the diauxic shift and respiratory growth. (E) Fluorescence values normalized to OD660 to account for changes in cell density are plotted across the growth curve for strains expressing YFP driven by *P*_*TDH1*_ (light blue, same data as panel D), *P*_*TDH2*_ (dark blue, same data as panel C), or *P*_*TDH3*_ (black, same data as panel B) promoters. The *TDH3* promoter drives expression at a much higher level (approx. 6x) than that of *P*_*TDH1*_ or *P*_*TDH2*_.
(PDF)

S1 File Primers and guide RNA target sequences used to generate engineered strains. Nucleotide sequences and brief descriptions of transformation protocols used to generate strains bearing fluorescent reporters and fluorescent fusion proteins under the control of various promoters.
(PDF)
