# Supplementary material for: Active compensation for changes in TDH3 expression mediated by direct regulators of TDH3 in Saccharomyces cerevisiae
Source: PLoS Genet. 2023 Dec 13;19(12):e1011078. doi: 10.1371/journal.pgen.1011078 (PMC10752532; doi:10.1371/journal.pgen.1011078)
Supplement: S1 Fig — (A) Population growth curves (measured using the optical density (OD) at 660 nm) are shown for strains expressing a yellow fluorescent protein (YFP) driven by PTDH1 (light blue), PTDH2 (dark blue), or PTDH3 (black). All three strains showed similar growth dynamics. Data in (A) was used to demarcate lag phase (red), exponential growth (green), diauxic shift (blue), and respiratory growth (purple) phases for all panels. Three replicates of each strain are shown. (B-D) Fluorescence values normalized to OD660 to account for changes in cell density are plotted across the growth curve for strains expressing YFP driven by PTDH3 (B), PTDH2 (C), and PTDH1 (D). PTDH3 and PTDH2 dynamics are similar across the stages of the growth curve (lag phase in red, exponential growth in green, diauxic shift in blue, and respiratory growth in purple, see panel A), with expression increasing during early exponential growth and then declining, halting during the diauxic shift, and increasing at a slower rate throughout respiratory growth. PTDH1 shows different dynamics; as TDH2 and TDH3 begin to decline, TDH1 begins to increase and does so steadily throughout the diauxic shift and respiratory growth. (E) Fluorescence values normalized to OD660 to account for changes in cell density are plotted across the growth curve for strains expressing YFP driven by PTDH1 (light blue, same data as panel D), PTDH2 (dark blue, same data as panel C), or PTDH3 (black, same data as panel B) promoters. The TDH3 promoter drives expression at a much higher level (approx. 6x) than that of PTDH1 or PTDH2. (PDF) [file pgen.1011078.s002.pdf]

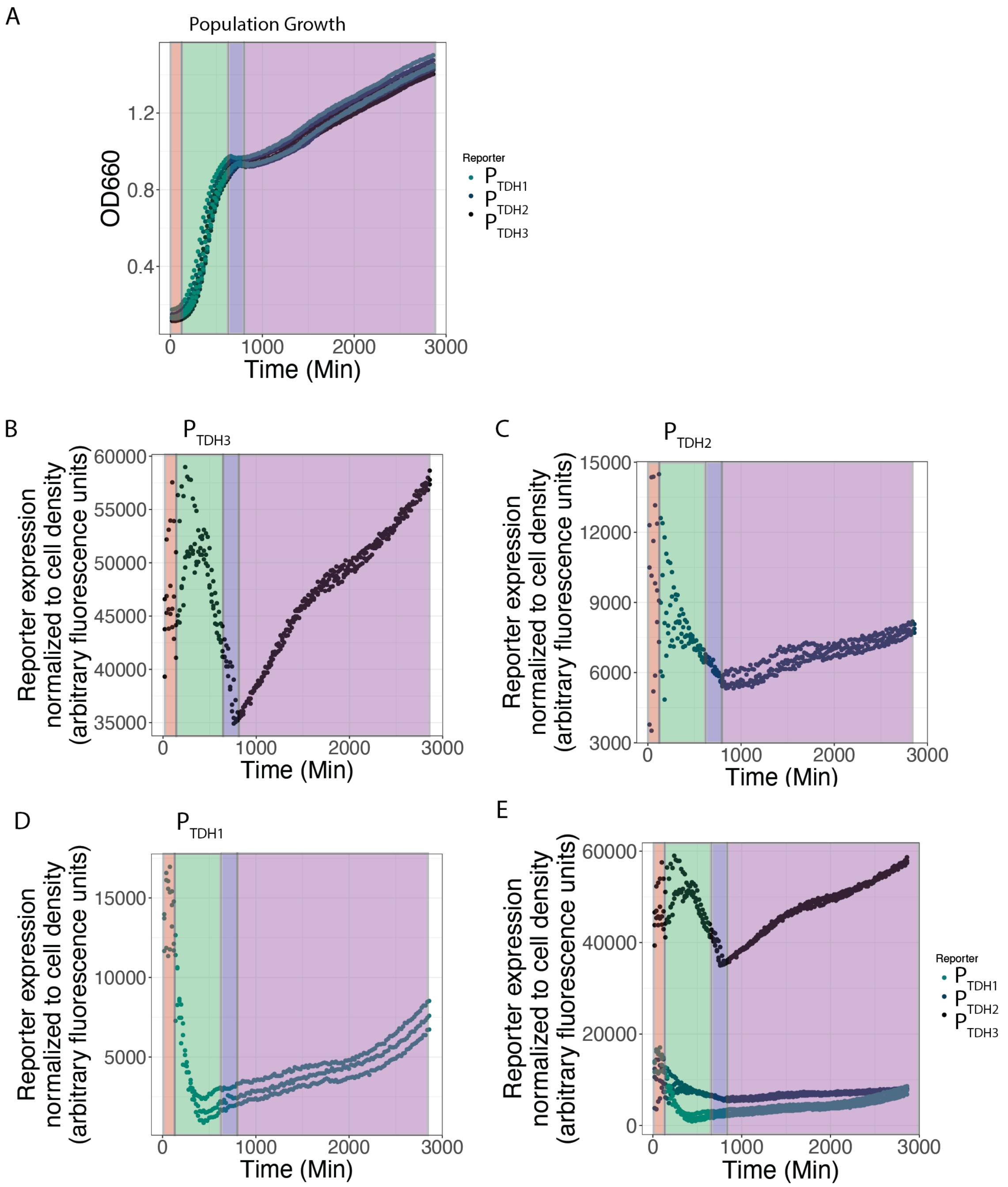

S1 Figure. *TDH1*, *TDH2*, and *TDH3* are expressed at different levels and under different growth conditions. (A) Population growth curves (measured using the optical density (OD) at 660 nm) are shown for strains expressing a yellow fluorescent protein (YFP) driven by P_{TDH1} (light blue), P_{TDH2} (dark blue), or P_{TDH3} (black). All three strains showed similar growth dynamics. Data in (A) was used to demarcate lag phase (red), exponential growth (green), diauxic shift (blue), and respiratory growth (purple) phases for all panels. Three replicates of

each strain are shown. (B-D) Fluorescence values normalized to OD660 to account for changes in cell density are plotted across the growth curve for strains expressing YFP driven by P_{TDH3} (B), P_{TDH2} (C), and P_{TDH1} (D). P_{TDH3} and P_{TDH2} dynamics are similar across the stages of the growth curve (lag phase in red, exponential growth in green, diauxic shift in blue, and respiratory growth in purple, see panel A), with expression increasing during early exponential growth and then declining, halting during the diauxic shift, and increasing at a slower rate throughout respiratory growth. P_{TDH1} shows different dynamics; as *TDH2* and *TDH3* begin to decline, *TDH1* begins to increase and does so steadily throughout the diauxic shift and respiratory growth. (E) Fluorescence values normalized to OD660 to account for changes in cell density are plotted across the growth curve for strains expressing YFP driven by P_{TDH1} (light blue, same data as panel D), P_{TDH2} (dark blue, same data as panel C), or P_{TDH3} (black, same data as panel B) promoters. The *TDH3* promoter drives expression at a much higher level (approx. 6x) than that of P_{TDH1} or P_{TDH2}.
