# Supplementary material for: Active compensation for changes in TDH3 expression mediated by direct regulators of TDH3 in Saccharomyces cerevisiae
Source: PLoS Genet. 2023 Dec 13;19(12):e1011078. doi: 10.1371/journal.pgen.1011078 (PMC10752532; doi:10.1371/journal.pgen.1011078)
Supplement: S1 File — Nucleotide sequences and brief descriptions of transformation protocols used to generate strains bearing fluorescent reporters and fluorescent fusion proteins under the control of various promoters. (PDF) [file pgen.1011078.s003.pdf]

S1 File: Primers and guide RNA target sequences used to generate engineered strains

Creation of TDH2::P^{TDH2}-CFP-TDH2 strains: To generate a strain with a fusion protein consisting of cyan fluorescent protein (CFP) fused to the N-terminus of TDH2 and driven by the native TDH2 promoter, we generated the fusion construct using strand overlap extension (SOE) PCR. We then introduced double-strand breaks at the native TDH2 locus using CRISPR-Cas9 as described in [1]. The sequence of the primers used for SOE PCR and 20bp sequence targeted with CRISPR are below:

SOE PCR Primers:

Right fragment F’: Primer3981:
CGGGTTAATTAACAGTGTTAGAGTTGCTATTAACGGTTTCGG
Right fragment R’: Primer3983:
TAGAGAAGCCCATGGCAAGTTAGC
Left fragment F’: Primer601:
GGTTGAAACCAGTTCCCTGA
Left fragment R’: Primer3985:
CTAACACTGTTAATTAACCCGGGGATCCGTCGACCTTTGTACAATTCATCCATACCTAG
SOE PCR F’: Primer3982:
CCAAGAACTTAGTTTCAAATTAAATTCATCACACAAACAAACAAAACAAAATGTCTAAAG
GTGAAGAATTATTCACTGG
SOE PCR R’: Primer3984:
GGGAAACTTCACCAGCGTATCTACC

Yeast Strains used as Templates (gDNA):

Left: YPW862
Right: YPW3016

TDH2_gRNAtarget: Plasmid612: CACAAACAAACAAAACAAAA

Creation of TDH3::ΔTDH3 strains: To delete TDH3, We induced double-strand breaks at the TDH3 locus with CRISPR-Cas9 and induced homology-mediated recombination with a repair fragment that had homology arms to the locus but lacked the protein-coding sequence. The CRISPR-Cas9 plasmid for deleting TDH3 contains was created via the restriction digestion and ligation method described in [1]. The reference strain was then transformed with the engineered plasmid and the repair fragment that guided site-specific homology-directed repair. The repair fragment contained homology arms flanking the TDH3 protein coding sequence. The sequence of the 20bp region targeted with CRISPR and of the repair fragment used to delete TDH3 are below:

TDH3_gRNAtarget: ACACACATAAACAAACAAAA

TDH3 deletion repair fragment:
CGGTAGGTATTGATTGTAATTCTGTAAATCTATTTCTTAAACTTCTTAAATTCTACTTTTATA
GTTAGTCCTTGATGCGCTATTGCATTGTTCTTGTCTTTTTCGCCACATGTAATATCTGTAG
TAGATACCTGATAC

Creation of HO::promoter-YFP reporter strains: We used a similar strategy as above to integrate promoter-YFP constructs into the HO locus. We used a strain that had a truncated

form of YFP that lacked the start codon, a promoter, and that had a CRISPR target sequence immediately upstream of the YFP. This strain lacked fluorescence. We then targeted double-strand breaks to this site using CRISPR-Cas9 and recombined in repair fragments containing a 5’ homology arm to the site of integration, the desired promoter variants, and an in-frame fused portion of YFP containing the start codon. Transformed strains were screened first for fluorescence and then were sequenced with Sanger Sequencing to verify proper integration. The specific sequences of the repair fragments are below. Note that the portion of YFP in the repair fragment both served as a homology arm and restored expression of the truncated YFP in our genomic integration site.

HO_gRNA target: AACAATGCAATAGCGCATCA

Repair fragments:
Colors highlight different sequence elements. (Gray highlight--5’ homology arm, blue – promoter, yellow highlight– beginning of YFP to restore expression and serve as 3’ homology arm. Regions bound by forward and reverse primers are underlined.)

>Ptdh2_GCRabol
CAAATCAGTGCCGGTAACGCTTTTTGTATCTTGAGATGGCGTATTTCTACTCCAGCATTCTAGTTAA
GAAAAAGTCTAAAAATGGTTTTTTTCATCCAAAATATTAAATTTTACTTTTATTACATACAACTTTTTAA
ACTAATATACACATTGTTAAGAAACGATATCACAACTGTTACGACAGAGAGAGACCCAAGCTAGAG
ATCACAAGCTAAAAAAGAACCAAGTTTACATATATATATATATATATATATATATATATATATATATCCATAT
TCATATTTCTCGAGAAAGAGCCTCTATTTCTCATTGGTAAGTAACTTCATAAGAGACTAAGTTGTAAA
ACTGTGGCTTTGTTATACGGTGATTTCCTTTGGAGGTTGCTAAGGTTTATGGTGTTGAGTGCAGTG
TGCACGACAGGGACCGCTAGAATGCGGTGAGTTACAAAATTACACGTGACTTTTCTGGTCACGTG
ACCTTTTTTTCTGTCAGCAATCCGTAGGATGCGCGTTGGCGCTACAAGTGTGTCATATCTGTACTA
TATTTGTACACTTATATGTAGTTGTGACAAAAGTCTCTGTTAGTACTAAATTAAACGATGTTATATCTG
TGGACCCCCTCACCTTATACCACTACGTACATATCGTTGGAAAATCTAGATCAGAGGGTGGTAAAT
GAAGTGTAATAGTATTCATTTTTCTTATAAATCATCCCTTCCGTGATTTATACAAAAGAAGAGGAGAA
TATGCTGAATACTTGGTATATTACTCTACATTATACTCTTATCTTGACGGGTATTCTGAGCATCTTACT
CAGTTTCAAGATCTTTTAATGTCCAAAAACATTTGAGCCGATCTAAATACTTCTGTGTTTTCATTAAT
TTATAAATTGTACTCTTTTAAGACATGGAAAGTACCAACATCGGTTGAAACAGTTTTTCATTTACTTA
TGGTTTATTGGTTTTTCCAGTGAATGATTATTTGTCGTTACCCTTTCGTAAAAGTTCAAACACGTTTT
TAAGTATTGTTTAGTTGCTCTTTCGACATATATGATTATCCCTGCGCGGCTAAAGTTACAAAAAACAT
AAGACAACTGAAGTTAATTTACGTCAATTAAGTTTTCCAGGGTAATGATGTTTTGGCTAATTCAATAA
GTATGTCATGAAATACGTTGTGAAGAGAAATAATGAAAAGAAACAACGAAACTGGGTCGGCCTGTT
GTTTCTTTTCTTTACCACGTGATCTGCGGCATTTACCGCGCGTTTTGCGCAGTTGTTGCAACGCA
GCTACGGCTAACAAAGCCTAGTGGAACTCGACTGATGTGTTAGGGCCTAAAACTGGTGGTGACA
GCTGAAGTGAACTATTCAATCCAATCATGTCATGGCTGTCACAAAGACCTTGCGGACCGCACGTA
CGAACACATACGTATGCTAATATGTGTTTTGATAGTACCCAGTGATCGCAGACCTGCAATTTTTTTG
TAGGTTTGGAAGAATATATAAAGGTTGCACTCATTCAAGATAGTTTTTTTCTTGTGTGTCTATTCATT
TTATTATTGTTTGTTTAAATGTTAAAAAAACCAAGAACTTAGTTTCAAATTAAATTCATCACACAAACA
AACAAAACAAAATGTCTAAAGGTGAAGAATTATTCACTGGTGTTGTCCCAATTTTGGTTGAATTAGA
TGGTGATGTTAATGGTCACAAATTTTCTGTCTCCGGTGAAGGTGAAGGTGATGCTACTTACGGTAA
ATTGACCTTAAAATTAATTTGTACTACTGGTAAATTGCCAGTTCCATGGCCAACCTTAGTCACTACTT
TAGGTTATGGT

>Ptdh2_GCRmut
CAAATCAGTGCCGGTAACGCTTTTTGTATCTTGAGATGGCGTATTTCTACTCCAGCATTCTAGTTAA
GAAAAAGTCTAAAAATGGTTTTTTTCATCCAAAATATTAAATTTTACTTTTATTACATACAACTTTTTAA
ACTAATATACACATTGTTAAGAAACGATATCACAACTGTTACGACAGAGAGAGACCCAAGCTAGAG
ATCACAAGCTAAAAAAGAACCAAGTTTACATATATATATATATATATATATATATATATATATATATCCATAT
TCATATTTCTCGAGAAAGAGCCTCTATTTCTCATTGGTAAGTAACTTCATAAGAGACTAAGTTGTAAA
ACTGTGGCTTTGTTATACGGTGATTTCCTTTGGAGGTTGCTAAGGTTTATGGTGTTGAGTGCAGTG

TGCACGACAGGGACCGCTAGAATGCGGTGAGTTACAAAATTACACGTGACTTTTCTGGTCACGTG
ACCTTTTTTTCTGTCAGCAATCCGTAGGATGCGCGTTGGCGCTACAAGTGTGTCATATCTGTACTA
TATTTGTACACTTATATGTAGTTGTGACAAAAGTCTCTGTTAGTACTAAATTAAACGATGTTATATCTG
TGGACCCCCTCACCTTATACCACTACGTACATATCGTTGGAAAATCTAGATCAGAGGGTGGTAAAT
GAAGTGTAATAGTATTCATTTTTCTTATAAATCATCCCTTCCGTGATTTATACAAAAGAAGAGGAGAA
TATGCTGAATACTTGGTATATTACTCTACATTATACTCTTATCTTGACGGGTATTCTGAGCATCTTACT
CAGTTTCAAGATCTTTTAATGTCCAAAAACATTTGAGCCGATCTAAATACTTCTGTGTTTTCATTAAT
TTATAAATTGTACTCTTTTAAGACATGGAAAGTACCAACATCGGTTGAAACAGTTTTTCATTTACTTA
TGGTTTATTGGTTTTTCCAGTGAATGATTATTTGTCGTTACCCTTTCGTAAAAGTTCAAACACGTTTT
TAAGTATTGTTTAGTTGCTCTTTCGACATATATGATTATCCCTGCGCGGCTAAAGTTAAGAATGCAAA
AAACATAAGACAACTGAAGTTAATTTACGTCAATTAAGTTTTCCAGGGTAATGATGTTTTGGGCTTT
CACTAATTCAATAAGTATGTCATGAAATACGTTGTGAAGAGCATTCAGAAATAATGAAAAGAAACAA
CGAAACTGGGTCGGCCTGTTGTTTCTTTTCTTTACCACGTGATCTGCGGCATTTACAGAAAGTCG
CGCGTTTTGCGCAGTTGTTGCAACGCAGCTACGGCTAACAAAGCCTAGTGGAACTCGACTGATG
TGTTAGGGCCTAAAACTGGTGGTGACAGCTGAAGTGAACTATTCAATCCAATCATGTCATGGCTGT
CACAAAGACCTTGCGGACCGCACGTACGAACACATACGTATGCTAATATGTGTTTTGATAGTACCC
AGTGATCGCAGACCTGCAATTTTTTTGTAGGTTTGGAAGAATATATAAAGGTTGCACTCATTCAAGA
TAGTTTTTTTCTTGTGTGTCTATTCATTTTATTATTGTTTGTTTAAATGTTAAAAAAACCAAGAACTTA
GTTTCAAATTAAATTCATCACACAAACAAACAAAACAAAATGTCTAAAGGTGAAGAATTATTCACTG
GTGTTGTCCCAATTTTGGTTGAATTAGATGGTGATGTTAATGGTCACAAATTTTCTGTCTCCGGTGA
AGGTGAAGGTGATGCTACTTACGGTAAATTGACCTTAAAATTAATTTGTACTACTGGTAAATTGCCA
GTTCCATGGCCAACCTTAGTCACTACTTTAGGTTATGGT

>Ptdh3_Rap1mut
CAAATCAGTGCCGGTAACGCTTTTTGTATCTTGAGATGGCGTATTTCTACTCCAGCATTCTAGTTAA
GAAAAAGTCTAAAAATGGTTTTTTTCATCCAAAATATTAAATTTTACTTTTATTACATACAACTTTTTAA
ACTAATATACACATTGTTCGAGTTTATCATTATCAATACTGCCATTTCAAAGAATACGTAAATAATTAAT
AGTAGTGATTTTCCTAACTTTATTTAGTCAAAAAATTAGCCTTTTAATTCTGCTGTAACCCGTACATG
CCCAAAATAGGGGGCGGGTTACACAGAATATATAACATCGTAGGTGTCTGAGTGAACAGTTTATTC
CTGGCATCCACTAAATATAATGGAGCCCGCTTTTTAAGCTGGCATCCAGAAAAAAAAAGAATCCCA
GCACCAAAATATTGTTTTCTTCACCAACCATCAGTTCATAGGTCCATTCTCTTAGCGCAACTACAGA
GAACAGGGGCACAAACAGGCAAAAAACGGGCACAACCTCAATGGAGTGATGCAACCTGCCTGG
AGTAAATGATGACACAAGGCAATTGACCCACGCATGTATCTATCTCATTTTCTTACACCTTCTATTAC
CTTCTGCTCTCTCTGATTTGGAAAAAGCTGAAAAAAAAGGTTGAAACCAGTTCCCTGAAATTATTC
CCCTACTTGACTAATAAGTATATAAAGACGGTAGGTATTGATTGTAATTCTGTAAATCTATTTCTTAAA
CTTCTTAAATTCTACTTTTATAGTTAGTCTTTTTTTTTAGTTTTAAAACACCAAGAACTTAGTTTCGAAT
AAACACACATAAACAAACAAAATGTCTAAAGGTGAAGAATTATTCACTGGTGTTGTCCCAATTTTGG
TTGAATTAGATGGTGATGTTAATGGTCACAAATTTTCTGTCTCCGGTGAAGGTGAAGGTGATGCTA
CTTACGGTAAATTGACCTTAAAATTAATTTGTACTACTGGTAAATTGCCAGTTCCATGGCCAACCTTA
GTCACTACTTTAGGTTATGGT

>Ptdh3_Gcr482mut
CAAATCAGTGCCGGTAACGCTTTTTGTATCTTGAGATGGCGTATTTCTACTCCAGCATTCTAGTTAA
GAAAAAGTCTAAAAATGGTTTTTTTCATCCAAAATATTAAATTTTACTTTTATTACATACAACTTTTTAA
ACTAATATACACATTGTTCGAGTTTATCATTATCAATACTGCCATTTCAAAGAATACGTAAATAATTAAT
AGTAGTGATTTTCCTAACTTTATTTAGTCAAAAAATTAGCCTTTTAATTCTGCTGTAACCCGTACATG
CCCAAAATAGGGGGCGGGTTACACAGAATATATAACATCGTAGGTGTCTGGGTGAACAGTTTATTC
CTGGCATTCACTAAATATAATGGAGCCCGCTTTTTAAGCTGGCATCCAGAAAAAAAAAGAATCCCA
GCACCAAAATATTGTTTTCTTCACCAACCATCAGTTCATAGGTCCATTCTCTTAGCGCAACTACAGA
GAACAGGGGCACAAACAGGCAAAAAACGGGCACAACCTCAATGGAGTGATGCAACCTGCCTGG
AGTAAATGATGACACAAGGCAATTGACCCACGCATGTATCTATCTCATTTTCTTACACCTTCTATTAC
CTTCTGCTCTCTCTGATTTGGAAAAAGCTGAAAAAAAAGGTTGAAACCAGTTCCCTGAAATTATTC
CCCTACTTGACTAATAAGTATATAAAGACGGTAGGTATTGATTGTAATTCTGTAAATCTATTTCTTAAA
CTTCTTAAATTCTACTTTTATAGTTAGTCTTTTTTTTTAGTTTTAAAACACCAAGAACTTAGTTTCGAAT
AAACACACATAAACAAACAAAATGTCTAAAGGTGAAGAATTATTCACTGGTGTTGTCCCAATTTTGG
TTGAATTAGATGGTGATGTTAATGGTCACAAATTTTCTGTCTCCGGTGAAGGTGAAGGTGATGCTA

CTTACGGTAAATTGACCTTAAAATTAATTTGTACTACTGGTAAATTGCCAGTTCCATGGCCAACCTTA
GTCACTACTTTAGGTTATGGT

Ptdh3_Gcr444mut
CAAATCAGTGCCGGTAACGCTTTTTGTATCTTGAGATGGCGTATTTCTACTCCAGCATTCTAGTTAA
GAAAAAGTCTAAAAATGGTTTTTTTCATCCAAAATATTAAATTTTACTTTTATTACATACAACTTTTTAA
ACTAATATACACATTGTTCGAGTTTATCATTATCAATACTGCCATTTCAAAGAATACGTAAATAATTAAT
AGTAGTGATTTTCCTAACTTTATTTAGTCAAAAAATTAGCCTTTTAATTCTGCTGTAACCCGTACATG
CCCAAAATAGGGGGCGGGTTACACAGAATATATAACATCGTAGGTGTCTGGGTGAACAGTTTATTC
CTGGCATCCACTAAATATAATGGAGCCCGCTTTTTAAGCTGGCATTCAGAAAAAAAAAGAATCCCA
GCACCAAAATATTGTTTTCTTCACCAACCATCAGTTCATAGGTCCATTCTCTTAGCGCAACTACAGA
GAACAGGGGCACAAACAGGCAAAAAACGGGCACAACCTCAATGGAGTGATGCAACCTGCCTGG
AGTAAATGATGACACAAGGCAATTGACCCACGCATGTATCTATCTCATTTTCTTACACCTTCTATTAC
CTTCTGCTCTCTCTGATTTGGAAAAAGCTGAAAAAAAAGGTTGAAACCAGTTCCCTGAAATTATTC
CCCTACTTGACTAATAAGTATATAAAGACGGTAGGTATTGATTGTAATTCTGTAAATCTATTTCTTAAA
CTTCTTAAATTCTACTTTTATAGTTAGTCTTTTTTTTAGTTTTAAAACACCAAGAACTTAGTTTCGAAT
AAACACACATAAACAAACAAAATGTCTAAAGGTGAAGAATTATTCACTGGTGTTGTCCCAATTTTGG
TTGAATTAGATGGTGATGTTAATGGTCACAAATTTTCTGTCTCCGGTGAAGGTGAAGGTGATGCTA
CTTACGGTAAATTGACCTTAAAATTAATTTGTACTACTGGTAAATTGCCAGTTCCATGGCCAACCTTA
GTCACTACTTTAGGTTATGGT

>Ptdh3_Gcr485mut
CAAATCAGTGCCGGTAACGCTTTTTGTATCTTGAGATGGCGTATTTCTACTCCAGCATTCTAGTTAA
GAAAAAGTCTAAAAATGGTTTTTTTCATCCAAAATATTAAATTTTACTTTTATTACATACAACTTTTTAA
ACTAATATACACATTGTTCGAGTTTATCATTATCAATACTGCCATTTCAAAGAATACGTAAATAATTAAT
AGTAGTGATTTTCCTAACTTTATTTAGTCAAAAAATTAGCCTTTTAATTCTGCTGTAACCCGTACATG
CCCAAAATAGGGGGCGGGTTACACAGAATATATAACATCGTAGGTGTCTGGGTGAACAGTTTATTC
CTGGTATCCACTAAATATAATGGAGCCCGCTTTTTAAGCTGGCATCCAGAAAAAAAAAGAATCCCA
GCACCAAAATATTGTTTTCTTCACCAACCATCAGTTCATAGGTCCATTCTCTTAGCGCAACTACAGA
GAACAGGGGCACAAACAGGCAAAAAACGGGCACAACCTCAATGGAGTGATGCAACCTGCCTGG
AGTAAATGATGACACAAGGCAATTGACCCACGCATGTATCTATCTCATTTTCTTACACCTTCTATTAC
CTTCTGCTCTCTCTGATTTGGAAAAAGCTGAAAAAAAAGGTTGAAACCAGTTCCCTGAAATTATTC
CCCTACTTGACTAATAAGTATATAAAGACGGTAGGTATTGATTGTAATTCTGTAAATCTATTTCTTAAA
CTTCTTAAATTCTACTTTTATAGTTAGTCTTTTTTTTAGTTTTAAAACACCAAGAACTTAGTTTCGAAT
AAACACACATAAACAAACAAAATGTCTAAAGGTGAAGAATTATTCACTGGTGTTGTCCCAATTTTGG
TTGAATTAGATGGTGATGTTAATGGTCACAAATTTTCTGTCTCCGGTGAAGGTGAAGGTGATGCTA
CTTACGGTAAATTGACCTTAAAATTAATTTGTACTACTGGTAAATTGCCAGTTCCATGGCCAACCTTA
GTCACTACTTTAGGTTATGGT

References

1. Laughery MF, Hunter T, Brown A, Hoopes J, Ostbye T, Shumaker T, et al. New vectors for simple and streamlined CRISPR-Cas9 genome editing in Saccharomyces cerevisiae. Yeast. 2015;32: 711–720.
