# Supplementary material for: Active compensation for changes in TDH3 expression mediated by direct regulators of TDH3 in Saccharomyces cerevisiae
Source: PLoS Genet. 2023 Dec 13;19(12):e1011078. doi: 10.1371/journal.pgen.1011078 (PMC10752532; doi:10.1371/journal.pgen.1011078)
Supplement: Attachment — Submitted filename: response to reviewers_rev.pdf [file pgen.1011078.s004.pdf]

Full Revision

Review COMMONS

Summary: The original manuscript submitted to *Review Commons* included only RNA-seq data, and the reviewers asked for complementary data validating key RNA-seq findings, including determining whether compensation is also seen at the protein level, and further testing of our hypothesis that specific transcription factors mediate compensatory changes in gene expression. They also wanted to know more about divergent regulation of the paralogous TDH genes. This revised manuscript includes new data addressing all of these points. Specifically, we have added:

- Expression of reporter genes in which the *TDH2* or *TDH3* promoter drives expression of the YFP coding sequence measured using flow cytometry. These reporter genes showed the same upregulation of expression in the absence of the native *TDH3* gene as seen in the RNA-seq data.
- Expression of a new *TDH2::CFP* fusion protein measured using flow cytometry that shows the upregulation of TDH2p protein in the absence of *TDH3*. This shows that the change in expression seen at the mRNA level in the RNA-seq data is also seen at the protein level.
- Expression of *TDH2* and *TDH3* YFP reporter genes in which the GCR1p and/or RAP1p transcription factor binding sites have been mutated. These mutants failed to show the compensatory response to deletion of the native *TDH3* gene that was seen with the wild-type *TDH2* and *TDH3* promoter alleles.
- A supplementary figure using reporter genes to show expression of *TDH1*, *TDH2*, and *TDH3* at different stages of the growth curve, illustrating how their expression and regulation has diverged.

A complete, point-by-point, response to the reviewers' comments follows.

2. Point-by-point description of the revisions

Review Commons prompts are in gray, reviewer comments are in black, and our responses are in purple.

Reviewer #1 (Evidence, reproducibility and clarity (Required)):
1. EVIDENCE, REPRODUCIBILITY AND CLARITY
==
Summary:
Provide a short summary of the findings and key conclusions (including methodology and model system(s) where appropriate). Please place your comments about significance in section 2.

This work examines the active compensation of TDH3 by its paralogs TDH1 and TDH2 as a mechanism of robustness against genetic perturbations in yeast. The authors demonstrate that the paralogs compensate in a dose-dependent manner in response to TDH3's absence, mediated by shared transcriptional regulators Gcr1p and Rap1p. Furthermore, other glycolytic genes regulated by Gcr1p and Rap1p show similar changes in expression, indicating that active compensation of TDH3 is part of a greater homeostatic feedback mechanism. Additionally, the authors suggest that the ability of paralogs to actively compensate for each other and contribute to genetic robustness is actively selected for or is simply a side effect of their ancestrally shared regulators with sensitivity to feedback mechanisms.

Major comments:
- Are the claims and the conclusions supported by the data or do they require additional experiments or analyses to support them?
The authors present robust evidence in this paper to substantiate their claims and conclusions. The comprehensive data provided effectively establishes a clear and compelling case for the role of active compensation among the TDH paralogs. I think that the authors' conclusions are well-supported with the data. Further experiments are not warranted at this time.

- Please request additional experiments only if they are essential for the conclusions. Alternatively, ask the authors to qualify their claims as preliminary or speculative, or to remove them altogether.
No need for further experiments to support the manuscript's conclusions at this time.

- If you have constructive further reaching suggestions that could significantly improve the study but would open new lines of investigations, please label them as "OPTIONAL".

Dear authors, I have a couple of experiments to open further lines of investigation:
Considering the modest expression level increase resulting from gene duplication of TDH3 (~35%), it may be worthwhile to further explore this phenomenon and its potential relationship with the limited availability of GRC1 and RAP1 transcription factors. It is conceivable that an attenuation mechanism could be involved in regulating TDH3 expression, and an examination of this possibility would provide valuable insights. An experimental approach utilizing a titratable promoter and assessment of mRNA and protein levels would offer a compelling means to probe this inquiry. (OPTIONAL).

The strain expressing *TDH3* at 135% of the wild-type expression level carries two copies of *TDH3,* but both copies have mutations in their promoter that reduce their individual expression relative to the wild-type alleles. We have clarified the text by adding "reducing expression levels from each promoter" on page 6, line 17.

The authors' discussion raises the question of whether the active compensation observed between the TDH paralogs is a result of selection or simply a consequence of their shared regulators. To address this question, one potential avenue for future research would be to test the ability of TDH1-2 gene products to compensate for the loss of TDH3 by expressing them under the TDH3 promoter, a stronger or an inducible promoter, and then, measuring the fitness of the resulting strains with a tdh3**Δ** background. This additional line of experimentation has the potential to improve our understanding of the regulatory networks involved and shed light on the selective pressures that contribute to the maintenance of these paralogs over evolutionary time. (OPTIONAL)

We agree that this question - how selection has acted on the catalytic activity of the three paralogous proteins in concert with their expression levels - is very interesting. In fact, experiments including those described by the reviewer are currently underway in the Wittkopp lab and will be the focus of a future manuscript.

- Are the suggested experiments realistic in terms of time and resources? It would help if you could add an estimated time investment for substantial experiments.
Not applicable.

- Are the data and the methods presented in such a way that they can be reproduced?
Yes.

- Are the experiments adequately replicated and statistical analysis adequate?
Yes.

Minor comments:

- Specific experimental issues that are easily addressable.
In the introduction of the manuscript (pp. 4 para. 1), it would be useful to provide a more comprehensive overview of the gene expression patterns and protein abundances of the three TDH paralogs. Including such information would better enable readers to understand the functional roles of these paralogs.

We have added a new figure (Figure S1) showing differences in expression levels and patterns across the growth curve for the three paralogs. In addition, we have added some discussion of the differences in the effects of *trans*-regulatory mutations on protein abundance of each paralog that was recently published by another group and further indicates some level of regulatory divergence, particularly for *TDH1* (pp.4, lines 12-19).

It would be helpful to report the phenotype of the tdh1**Δ**/tdh2**Δ** double mutant to provide a clearer understanding of the functional overlap of these paralogs.

The revised manuscript includes additional information about divergence in expression patterns and differences in the effects of *trans*-regulatory mutations between *TDH1* and the other two paralogs. Specifically, *TDH1* is expressed under different conditions, and it is likely involved in different processes, than *TDH2* and *TDH3* (pp.4, lines 12-19, Figure S1). We have also added a sentence to the introduction stating that the double mutant deleting *TDH1* and *TDH3* has the same growth rate as *TDH3* mutants alone, suggesting that *TDH1* does not compensate for loss of *TDH3* in the same way that *TDH2* does. Because of these observations and because of the stronger overlap in expression profiles of *TDH2* and *TDH3*, we have chosen to focus primarily on the compensation for *TDH3* by *TDH2* in

the revised manuscript. We believe that these changes make the *TDH1/TDH2* double mutant phenotype (which has not been studied as closely as the double mutants of *TDH1* or *TDH2* with *TDH3*) unnecessary for this study.

In the results section (pp. 5, para. 2), while it is understandable that the authors have focused on the transcriptional regulation of these paralogs, it would also be insightful to provide data on their respective protein abundances, as posttranslational regulation is often a crucial component of gene expression. This data may already be available in other high-throughput studies.

We have added new experimental data using fluorescent fusion proteins that shows that the protein abundance of *TDH2* increases in response to deletion of *TDH3* (Figure 1B). The results of our fluorescence measurements correspond well with transcriptional levels indicated by RNA-seq, indicating that the upregulation of *TDH2* expression we saw in *TDH3* mutants was controlled primarily at the transcriptional level.

It would be valuable to include more detailed information on the shared cis-regulatory elements between these genes, as this could provide further insight into their regulation and potential functional divergence.

According to experimental data compiled in http://www.yeastract.com/ , ChIP-exo data indicates that promoters for *TDH1*, *TDH2*, and *TDH3* are all directly bound by Gcr1p (and the complex partner Gcr2p), although the evidence for Gcr1p binding is weaker at *TDH1* than the other two paralogs, and this study does not identify Gcr1p TFBS motifs in the promoters of either *TDH1* or *TDH2* (Holland et al. 2019). However, we were able to locate Gcr1p TFBS motifs (CTTCC, Baker 1991) in the *TDH2* promoter by manually searching regions annotated as bound by Gcr1's complex partner Gcr2p in another publicly available ChIP-chip dataset (MacIsaac et al. 2006). We mutated these four motifs in a copy of the *TDH2* promoter driving YFP expression to test for their role in upregulation using flow cytometry. We found that mutation or deletion of these putative TFBS reduced the overall activity of the promoter, indicating that these sequences are functional, and also observed that upon mutation or deletion of these putative TFBSs reduced the upregulation of *TDH2* when *TDH3* was deleted (Figure 3E). A schematic of the *TDH2* promoter has been added to Figure 3 describing these experiments.

- Are prior studies referenced appropriately?
Yes.

- Are the text and figures clear and accurate?
The language used in this manuscript is clear and concise, making the material easily comprehensible to readers of various levels of expertise. The figures have a good quality for the most part and effectively complement the text to aid in the understanding of their findings.

- Do you have suggestions that would help the authors improve the presentation of their data and conclusions?
I have a few minor suggestions regarding your manuscript's figures:
In figures 1-3, it would be helpful to indicate the number of biological or technical replicates used for the statistical analyses displayed in the plots.

We have added the number of biological replicates for each genotype to our figure legends.

Please consider adding a sentence to the figure legends indicating that the raw data was generated in a previous study.

We have added a sentence indicating that the raw data was generated in a previous study to all relevant figure legends.

Figure 4E may benefit from alternative visualization methods, such as using lines or a different type of plot, to make it easier to distinguish each dataset.

In response to this and other reviewer comments, we have re-formatted Figure 4 to reduce the number of genes displayed in Figure 4E. We believe this greatly increases the readability of the figure and thank the reviewer for their suggestion.

Full Revision

Reviewer #1 (Significance (Required)):

2. SIGNIFICANCE
===============

- General assessment: provide a summary of the strengths and limitations of the study. What are the strongest and most important aspects? What aspects of the study should be improved or could be developed?

The study is noteworthy for its comprehensive analysis of previously reported data, offering a new understanding of the mechanisms behind the observed robustness of eukaryotic organisms, in particular the active compensation of TDH3 expression. The evidence presented in support of their conclusions is compelling. However, further research is required to investigate the role of active compensation at different regulation levels, in other paralogs, and under different environmental conditions.

- Advance: compare the study to the closest related results in the literature or highlight results reported for the first time to your knowledge; does the study extend the knowledge in the field and in which way? Describe the nature of the advance and the resulting insights (for example: conceptual, technical, clinical, mechanistic, functional,...).

This study provides new insights into the mechanisms of active compensation for the loss of gene expression in yeast. The authors demonstrate that the paralogs TDH1 and TDH2 upregulate in a dose-dependent manner in response to reductions in TDH3, mediated by shared transcriptional regulators Gcr1p and Rap1p. Furthermore, other glycolytic genes regulated by Rap1p and Gcr1p show similar changes in expression, indicating that active compensation of TDH3 by its paralogs is part of a larger homeostatic response. This study provides a mechanistic understanding of active compensation for the loss of gene expression in yeast and has potential implications for other organisms.

- Audience: describe the type of audience ("specialized", "broad", "basic research", "translational/clinical", etc...) that will be interested or influenced by this research; how will this research be used by others; will it be of interest beyond the specific field?

This study may attract a broad audience, as it provides insight into the mechanisms of active paralogous compensation. Their findings have potential implications beyond the yeast's specific field, as they may provide insight into the mechanisms of robustness in other genes and organisms. This research may be of interest in the fields of molecular biology and evolution in particular gene regulation.

- Please define your field of expertise with a few keywords to help the authors contextualize your point of view. Indicate if there are any parts of the paper that you do not have sufficient expertise to evaluate.

My field of expertise is molecular biology and evolution, specifically in the areas of gene duplication, gene expression and regulation, protein evolution, and interaction networks. I am familiar with some of the topics discussed in the paper, such as gene expression and regulation, and have a good understanding of the research related to these topics.

We thank the reviewer for their insightful comments and thorough reading of the manuscript. We believe that the revisions, as described in more detail below, improve the manuscript and we greatly appreciate the suggestions.

Reviewer #2 (Evidence, reproducibility and clarity (Required)):

The ms uses RNAseq data on S cerevisiae with TDH3 perturbations (cis and trans) from prior publication to look into RNA expression of TDH3 paralogues and genes within the same pathway. Analysis of both cis and trans TDH3 perturbation data suggests that the compensatory mechanisms (via either the paralogues or the upstream/downstream enzymes of the glycolytic pathway) are dependent on GCR1 and RAP1 transcription factors.

Major comment but OPTIONAL: The RNAseq data presented here convincingly convey the authors claims. Nevertheless, if any of the following data becomes available in the meantime, they will add a lot to the current ms: 1.

Protein expression data can independently validate the findings and help support/clarify potential issues emerging from the data on the glycolytic pathway - see 2nd minor comment.

The revised manuscript includes new data showing increased expression of *TDH2* upon deletion of *TDH3* at the protein level using a *TDH2:CFP* fusion protein under the control of the native *TDH2* promoter and at the native locus (Figure 1B). These protein-level data do indeed independently validate our RNA-seq findings for *TDH2*. We have also re-arranged Figure 4 and clarified the section of the manuscript describing changes in expression in the rest of the glycolytic pathway to better communicate that these changes in gene expression may or may not be part of an active compensation mechanism (see further discussion below).

2. Any data that show expression of TDH3 as a result of TDH1/TDH2 expression changes occurring independently of Gcr1/Rap1 can support the claims on robustness as a consequence of multiple paralogues being around.

We have RNA-sequencing data for strains in which *TDH1* or *TDH2* was deleted individually (GSE175398, data from Vande Zande et al., 2022). We saw that in these strains *TDH3* expression was not significantly increased. We believe that this finding is most likely due to the difference in basal expression levels between paralogs. *TDH3* is expressed at approximately 6x the level of *TDH2*, and *TDH1* is expressed in stationary phase rather than exponential growth as *TDH2* and *TDH3* are (See new supplementary Figure S1). Deletion or reduction of *TDH3* expression represents a much larger change in total GAPDH levels in the cell, and therefore might elicit a much stronger compensation response than deletion of *TDH2* or *TDH1*. We are interested in how the different expression levels, patterns, and enzymatic activity levels have diverged between paralogs and contribute to their relative function in the cell, and, as mentioned above, another member of the Wittkopp lab is currently working on a manuscript addressing these questions in greater detail. For these reasons, we have chosen not to include these data in the current manuscript.

Minor comments
1) Introduction and analysis framing: there seems to be two aspects for robustness and compensation that the manuscript focuses on. The one is through paralogues and the other via alteration in the expression of genes in the same pathway. The study shows both, yet there is particular weight on the paralogues.
The introduction should also mention both in a coherent and organized way. As an example, the second paragraph in the intro refers to 'upregulation of a paralog' in the 1st sentence, then it refers to an example that fits better to compensation through changes in expression of enzymes in the same pathway.

We have adjusted the language in the second paragraph of the introduction to clarify that the other enzymes that are actively compensating for *CLV1* or *SlCLV3* loss in arabidopsis and tomato are paralogs (pg.2 line 21- pg.3 line 8). In addition, we have adjusted our wording of the final introduction paragraph (pg. 5, lines 11-18), and the final results section (pg. 13, lines 17-23) to better communicate that the other genes are changing as part of a homeostatic response programmed into the regulatory network and may or may not contribute to fitness gains in a *TDH3* mutant.

2)Figure 4 results/Discussion: Not unexpectedly, PFK1 and PFK2 (in panels 4D and 4B) have very similar expression profiles with respect to TDH3 expression. (Considering that they are part of the same complex, one would expect that their expression levels should correlate at the very least). Yet, PFK1 did not make the significance cutoff. That can be misleading, so it warrants a comment, either on the respective results section or discussion. For that reason and to make easier comparisons expression data should be shown on the same panel (consolidate 4B and 4D) with significance annotations. It would also be nice to see some commentary in terms of pathway output upon changes in TDH3 expression. It seems as if there is a 'diffused signal' through the whole pathway that compensates for TDH3 perturbations, meaning all enzymes may be compensating to different degrees.

We appreciate these suggestions and have used them to re-arrange Figure 4 to more clearly show the response of genes that function at each step in the glycolytic pathway. As noted in the reviewer's comment, PFK1 and 2 are nearly identical in their expression profiles. The reason one is not significantly upregulated is that it has a higher variance among replicates than the other, which we now point out explicitly in the figure legend. By grouping these genes together in Figure 4, the similarity of their expression changes is much more obvious.

Reviewer #2 (Significance (Required)):

The study demonstrates an example of paralog-dependent changes in gene expression that contribute to phenotypic robustness. The active paralog compensation is transcription factor-dependent, and the same transcription factors

are also responsible for compensatory changes in expression levels of genes in the same pathway. I believe that this is an interesting case showing how a negative feedback mechanism in place to maintain pathway output and contribute to phenotypic robustness, receives and integrates signaling from different components of a pathway, including paralogues. The study relies solely on RNAseq data. Although convincing, protein expression data not only could validate the RNAseq data, but also could give a more accurate view of the respective expression profiles. The study describes a molecular mechanism in pathway regulation with broad interest in basic research. It also has particular interest with respect to paralog evolution and brings up questions on the forces that drive paralog divergence.

We appreciate this reviewer's comments and suggestions and have added several new figures that use fluorescent fusion proteins to provide a quantitative readout of protein expression levels. Specifically, we have added panels showing increased protein expression of TDH2 fused with CFP upon deletion of *TDH3* (Figure 1B). We have also added expression of fluorescent reporter genes driven by the *TDH1*, *TDH2*, and *TDH3* promoters showing the differences in their expression profiles across population growth stages (Supplementary Figure 1). Finally, we have analyzed fluorescent reporter genes with promoters containing mutated Gcr1p TFBS, which also suggest a dependence of the compensatory upregulation on GCR1p (Figures 2D, 3E).

Reviewer #3 (Evidence, reproducibility and clarity (Required)):

In their preprint titled "Active compensation for changes in TDH3 expression mediated by direct regulators of TDH3 in Saccharomyces cerevisiae" Zande and Wittkopp attempt to delineate the molecular mechanism behind compensation. They have chosen 3 paralogs, TDH1, 2 and 3 in Saccharomyces cerevisiae as their system of choice, building on an earlier study where they have used RNA-seq transcriptomics to characterize how global gene expression is affected by strains harbouring regulatory mutations at the TDH3 locus or a TDH3 gene knockout. The major claims made by the authors in this study are as follows:
i. The TDH1/2/3 system demonstrates compensation, such that changes in levels of expression of TDH3 result in altered expression levels of TDH1 and TDH2
ii. The mechanism of this compensation is "active", that is it involves modulating the transcription of paralogs in response to altered TDH3 in the cell.
iii. The transcription factors Gcr1 and Rap1 are likely candidates mediating this compensation. The effects of these regulators on TDH1 and TDH2 differ and produces different profiles of compensatory expression for these two genes.
iv. Since Gcr1 and Rap1 regulate other genes coding for glycolytic enzymes, compensation is related to altered expression of a larger cohort of genes.

Major comments:

1. The authors' claims regarding the roles of Rap1 and Gcr1 as mechanisms of compensation are supported by correlative evidence from RNA seq data. To establish the causal relationships that the authors intend, more directed experiments like the ones listed below are required:

i. Monitoring the activity of TDH1 and TDH2 promoters (as YFP-fused reporters) in the various strains

We have added new experimental data to the manuscript monitoring the activity of the *TDH1* and *TDH2* promoters driving YFP in different phases of the growth curve, demonstrating the divergence in their gene expression patterns (Supplementary Figure 1). Because of the divergence in expression patterns, we chose to focus additional efforts on *TDH2* and have added new data showing an increase in expression of a CFP::TDH2 fusion protein upon deletion of *TDH3*. These new experiments provide strong evidence of the causal relationship between the deletion of *TDH3* and an increase in *TDH2* expression.

ii. Generating mutant promoter reporters for TDH1, 2 and 3 that are unable to bind to Gcr1 and Rap1 and testing their activity in the various mutant strains

We have added new experimental data to the manuscript demonstrating that increases in the activity of the *TDH3* and *TDH2* promoters upon deletion of *TDH3* are dependent upon Gcr1p transcription factor binding sites, as originally hypothesized. Specifically, the new figure panel 3E consists of flow cytometry data showing that the *TDH2* promoter driving YFP expression increases in fluorescence upon deletion of *TDH3*, but that a comparable increase

does not occur when Gcr1p TFBSs in the *TDH2* promoter are mutated. In addition, the new figure panel 2D shows that the *TDH3* promoter driving YFP no longer increases in activity when a Gcr1 TFBSs is mutated. These new experiments provide strong evidence for the dependence of active compensation by upregulation upon shared transcription factor GCR1.

2. The authors claim that Gcr1 and Rap1 have similar impact on other glycolytic enzymes. However, these conclusions are also based on RNA -seq data and hence remain correlative. Based on the presented results alone, and lack of a molecular mechanism for why the levels of Rap1 and Gcr1 change in TDH3 mutant strains, it may just as easily be argued that the change in expression of other glycolytic enzymes (and therefore glycolytic flux) may be the cause for altered Rap1/Gcr1 activity and not the consequence. To test which of these possibilities are true, I would recommend the following approaches:
i. Promoter reporters for glycolytic enzymes of interest, and mutant versions that don't respond to Rap1/Gcr1
ii. Change glycolytic flux by altering growth conditions (e.g. fermentable/non-fermentable carbon source) and check to see if compensation is altered

While it is possible that mutations in the *TDH3* promoter that change *TDH3* expression alter the expression of other glycolytic genes, and this in turn alters Rap1/Gcr1 activity, resulting in the upregulation of the TDH paralogs, we believe it is more likely that changes in the activity of Rap1/Gcr1 are a cause rather than a consequence of altered expression of glycolytic genes because it has previously been shown that these genes are under the control of Rap1 and Gcr1. We have adjusted the wording of the final results section, and throughout the paper, to clarify that we believe the similar expression patterns observed for other glycolytic genes suggest that the increase in paralog expression that results in active compensation is part of a larger regulon, which indeed may be responsive to changes in glycolytic flux. We cannot say, however, whether the upregulation of other glycolytic genes is part of the compensatory response per se. We believe this clarification, in addition to the new experiments showing the dependence of *TDH3* and *TDH2* upregulation on transcription factor binding sites for GCR1, addresses the issues raised above.

Reviewer #3 (Significance (Required)):

This study attempts to address the mechanistic basis for an important homeostatic mechanism, i.e. compensation. Compensation is an almost universal mechanism seen in pathways with genetic redundancy. As pointed out by the authors, compensation ensures that gene regulatory networks produce robust outcomes and are resistant to perturbation. Though compensation is often observed, the mechanistic basis is usually unclear. This study throws light on possible transcriptional mechanisms that orchestrate compensation by altering expression levels of paralogous enzymes. In this regard the study is novel, important, and fills a lacuna in the area. However, in its current form, the study lacks the necessary causal evidence needed to substantiate the claims made by the authors. Further, the mechanism linking transcriptional regulation and metabolic flux is still lacking. As a result, though interesting, the study doesn't provide a complete picture and fails to make an impact.

We thank the reviewer for their comments and believe that the additional experiments and data added to the revised manuscript, including using fluorescent reporter genes and mutant alleles to measure the activity of promoters and show their dependence on RAP1/GCR1 binding sites, provide the causal evidence necessary to make this an impactful study.

Since I am not a yeast geneticist, it is possible that several of the concerns raised by me are due to my lack of knowledge of the system and some of the links that I find missing may have been demonstrated by others. If this is the case, I would suggest that the authors provide adequate background to address these concerns in the manuscript itself. It is my opinion, that this study, once shored up, will be of interest to a wide-readership and could also provide important experimental data that could be used for mathematical modeling.

We appreciate the reviewer's comments and believe that the changes we've made to the manuscript, including the addition of critical new data that complements and supports the RNA-seq data originally presented in the manuscript, does indeed make this a study that will be of interest to a wide readership.
